# Impact of cannabinoids on synapse markers in an SH-SY5Y cell culture model

**DOI:** 10.1038/s41537-024-00498-6

**Published:** 2024-10-25

**Authors:** Kirsten Jahn, Nina Blumer, Caroline Wieltsch, Laura Duzzi, Heiko Fuchs, Roland Meister, Adrian Groh, Martin Schulze Westhoff, Tillmann Horst Christoph Krüger, Stefan Bleich, Abdul Qayyum Khan, Helge Frieling

**Affiliations:** 1 Laboratory of Molecular Neurosciences, Department of Clinical Psychiatry, Medical School Hannover, Hanover, Germany; 2 Laboratory for Experimental Eye Research, Department of Ophthalmology, Medical School Hannover, Hanover, Germany; 3 Department of Clinical Psychiatry, Division of clinical psychology and sexual medicine, Medical School Hannover, Hanover, Germany; 4 Center for Systems Neurosciences Hannover, Hanover, Germany

**Keywords:** Molecular neuroscience, Cellular neuroscience

## Abstract

Patients suffering from schizophrenic psychosis show reduced synaptic connectivity compared to healthy individuals, and often, the use of cannabis precedes the onset of schizophrenic psychosis. Therefore, we investigated if different types of cannabinoids impact methylation patterns and expression of schizophrenia candidate genes concerned with the development and preservation of synapses and synaptic function in a SH-SY5Y cell culture model. For this purpose, SH-SY5Y cells were differentiated into a neuron-like cell type as previously described. Effects of the cannabinoids delta-9-THC, HU-210, and Anandamide were investigated by analysis of cell morphology and measurement of neurite/dendrite lengths as well as determination of methylation pattern, expression (real time-qPCR, western blot) and localization (immunocytochemistry) of different target molecules concerned with the formation of synapses. Regarding the global impression of morphology, cells, and neurites appeared to be a bit more blunted/roundish and to have more structures that could be described a bit boldly as resembling transport vesicles under the application of the three cannabinoids in comparison to a sole application of retinoic acid (RA). However, there were no obvious differences between the three cannabinoids. Concerning dendrites or branch lengths, there was a significant difference with longer dendrites and branches in RA-treated cells than in undifferentiated control cells (as shown previously), but there were no differences between cannabinoid treatment and exclusive RA application. Methylation rates in the promoter regions of synapse candidate genes in cannabinoid-treated cells were in between those of differentiated cells and untreated controls, even though findings were significant only in some of the investigated genes. In other targets, the methylation rates of cannabinoid-treated cells did not only approach those of undifferentiated cells but were also valued even beyond. mRNA levels also showed the same tendency of values approaching those of undifferentiated controls under the application of the three cannabinoids for most investigated targets except for the structural molecules (NEFH, MAPT). Likewise, the quantification of expression via western blot analysis revealed a higher expression of targets in RA-treated cells compared to undifferentiated controls and, again, lower expression under the additional application of THC in trend. In line with our earlier findings, the application of RA led to higher fluorescence intensity and/or a differential signal distribution in the cell in most of the investigated targets in ICC. Under treatment with THC, fluorescence intensity decreased, or the signal distribution became similar to the dispersion in the undifferentiated control condition. Our findings point to a decline of neuronal differentiation markers in our in vitro cell-culture system under the application of cannabinoids.

## Introduction

According to the current knowledge, schizophrenia is mainly a connectivity alteration^1^, with an emphasis on frontal and cingulate cortices^2^. Both brain areas are involved in information processing, which is disturbed in schizophrenia^2–4^. A loss of synaptic density of about 30% during adolescence in the dorsolateral prefrontal cortex is considered to be physiological, but patients suffering from schizophrenic psychosis show a higher reduction of about 60%^5^. There has been some evidence in the past showing a direct correlation between the use of cannabis and the onset of schizophrenic psychosis^6–8^. Therefore, we were interested in whether cannabinoids can alter synapse structure or function. To investigate potential alterations of morphology, synaptic structures, and function, we used our earlier established neural cell culture and characterization system^9^. The system is especially useful for the investigation of synapse markers as SH-SY5Y cells differentiated by 50 µM RA show a very clear arrangement and visibility of important neuronal parameters like neurites, branches, and growth cones with hardly any interferences by other cell types like in primary neuronal cell culture. Furthermore, SH-SY5Y cells are of human origin. Therefore, this rather simple cell line can be of great advantage for the basic research of synapse components on a molecular level. Furthermore, our characterization system implies a comprehensive set of synapse markers (concerned with the development, preservation, and function of synapses), which are investigated regarding their Deoxyribonucleic acid (DNA) methylation pattern, expression (ribonucleic acid (RNA)—and protein level) and localization on a cellular level. Epigenetic alterations (especially DNA methylation) are often linked to how environmental conditions can impact the readability of DNA sequences in the long term although the DNA sequence is not affected itself. There is already some evidence showing that cannabinoids can either directly (by alteration of the activity of DNA methylation regulating enzymes) or indirectly (by modulation of other neurotransmitter systems and thereby their downstream signal cascades) influence DNA methylation patterns in general^10^. In the past, we also investigated the methylation level of four of the target genes in therapy-resistant schizophrenic patients. It turned out that neurexin (NRXN1) and microtubule-associated protein tau (MAPT) methylation levels were significantly higher in those patients who consumed tetrahydrocannabinol (THC^11^).

In the current study, we decided to investigate the psychoactive cannabinoid delta-9-THC as well as Anandamide and Hu-210 (Hebrew University, substance 210) as main representatives for each of the three cannabinoid groups, i.e., exogenous phytocannabinoids, endogenous cannabinoids, and synthetic cannabinoids. Anandamide was included to figure out if this endogenous ligand of cannabinoid receptors, as part of the endocannabinoid system, would have less potentially aversive effects compared to the exogenous substance THC or even supportive effects on synapses, as it is known that the endocannabinoid system plays an essential role in the development of the nervous system^12^ and is a modulator of neurotransmission^13^. THC and Anandamide are relatively weak agonists^14,15^, and THC, as a partial agonist, can theoretically act as an antagonist. HU-210 is a highly potent synthetic pure agonist with an 80- to 800-fold higher receptor- affinity than delta-9-THC^15^. It was included to find out if potential effects depend on receptor affinity. Furthermore, some neuroprotective effects have been ascribed to this component and its enantiomer HU-211 in the past^16^.

The synaptic targets under investigation are described below in more detail and in relation to schizophrenia to illustrate their relevance to the design of our study.

Receptors for neurotransmitters are part of functional synapses. According to the current knowledge, most neurotransmitter systems are involved in the pathogenesis of schizophrenia, with an emphasis on the dopaminergic system^17^. At least, it is still thought to be one of the major contributors at the endpoint of the pathophysiological cascade with a fully developed symptom complex, as elevated dopamine levels belong to the most consistently reported neurochemical abnormalities in schizophrenic patients^18^. Furthermore, drugs that block dopamine D2 receptors are very effective in treating positive symptoms of schizophrenia^19^, and most neuroleptic drugs block this type of receptor^20^. Dopamine receptor type 2 (DRD2) is the most abundant Dopamine receptor in the brain (along with DRD1) and occurs pre- and postsynaptically.

As part of the (excitatory) glutamatergic system, which is, among other functions, essential for the maturation of synapses^21,22^, the ionotropic ligand-gated N-methyl-d-aspartate (glutamatergic NMDA receptor—GRIN) and non-NMDA-/kainate (glutamatergic kainate receptor—GRIK) were analyzed in the current study to include the most important glutamate receptor subfamilies.

As we were interested in the effects of different cannabinoids, the cannabinoid receptor 1 (CNR1) was also studied as part of the endocannabinoid system. CNR1 is mainly expressed in the brain, whereas cannabinoid receptor 2 (CNR2) is mainly localized on immune cells^23^. Therefore, CNR2 was not investigated in the current study. The endocannabinoid system also plays a vital role during brain development as it exerts a high impact on synaptogenesis, dendrite formation, and inter-neuronal migration^24^.

Besides the neurotransmitter receptors, certain “cofactor” genes are prerequisites for functional neurotransmission, like components of the cell skeleton, molecules important for neuron-neuron interaction, as well as certain intra- and extracellular factors, which are involved in dendrite outgrowth.

The specific components of the neuronal cell skeleton built the basis for the formation and stability of cell shape and extensions and are, therefore, the spatial prerequisites for cell-cell- contacts in terms of synapses. Altered lengths of Neurofilaments have been linked to schizophrenia in the past^25^. The group of neuron-specific components of the cell skeleton investigated in the present study comprised neurofilament-H (NEFH) as an intermediate filament and βIII-tubulin (TUBB3) as a microtubule as well as Microtubule-associated protein 2 (MAP2) which is especially important to maintain dendritic structure by its interaction with microtubules. In the past, a loss of MAP2 immuno-reactivity was shown across several cortical regions in schizophrenia (Shelton et al.^27^). Furthermore, we also investigated MAPT. Though it is mainly known to play a role in dementia, altered genetics of MAPT have also been linked with an increased risk of developing schizophrenic psychoses^26–29^.

Additionally, we monitored some molecules important for neuron-neuron interaction like postsynaptic density 95 (DLG4, PSD 95), synaptophysin (SYP), neuregulin-1 (NRG1), and neurexin (NRXN1).

DLG4 (PSD95) is known to play an important role in the clustering of receptors. In the past, there have been hints of altered expression of DLG4 in schizophrenia^30^. The release of neurotransmitters at the synaptic cleft is regulated by several molecules that interact in a highly sophisticated way. In the initiation of neurotransmission, the soluble N-ethylmaleimide-sensitive-factor attachment receptor (SNARE) complex (composed of synaptosomal-associated protein 25 kDa (SNAP-25), syntaxin, and synaptobrevin) plays an important role. Further acceleration of this process is typically achieved by synaptotagmin. For termination of transmission, synaptophysin (SYP) builds a complex with synaptobrevin, thereby inactivating it. As SYP modulates the efficiency of synapses also during development, it is therefore often used as an indicator of synaptic plasticity^31^. SYP is directly related to synaptic function and turnover of neurotransmitters as it regulates the trafficking of synaptobrevin and its retrieval after vesical fusion^32^ in virtually all neurons and seems to be of special importance during periods of increased and repetitive synaptic vesicle turnover^33^. Many reports could show significantly reduced levels of SYP in the prefrontal cortex (PFC) of schizophrenic patients^34–38^. Therefore, out of this group of molecules necessary for the regulation of neurotransmitter release, SYP was chosen as representative. Neuregulin (NRG1) is expressed presynaptically and interacts with its postsynaptic receptor erbB4 (human epidermal growth factor receptor 4, a receptor tyrosine-protein kinase), thereby leading to differentiation and synapse formation. NRG1 has been included in the group of schizophrenia risk genes in the past (for review, see Buonanno^39^). The interacting molecules neurexin (presynaptic) and neuroligin (postsynaptic) are important neuron–neuron adhesion molecules directly at the synapse^40^. Besides cell adhesion, especially NRXN1 has further functions such as the modulation of calcium channels, thereby also influencing the release of neurotransmitters^41^. Deletion in the NRXN1 gene has already been linked to the pathogenesis of schizophrenia^42^. Of the latter two pairs of interactors, NRG1 and NRXN1 were therefore chosen as representatives. The neural cell adhesion molecule (NCAM1) also mediates cell–cell adhesion in dependence on its sialyation status and has a high impact on neuritogenesis^43,44^. Elevated NCAM protein levels after differentiation with 10 µM RA have been shown before^45^, and an altered expression of NCAM has been associated with an increased risk of schizophrenia^46,47^. As the binding capacities of NCAM depend on glycosylation with polysialic acid, we also investigated the two polysialic acid transferases 2 (alpha-2,8-sialyltransferase 8b isoform 1/2 precursor, Chr. 15, ST8SIA2), and 4 (cmp-n-acetylneuraminate-poly-alpha-2,8-sialtransferase, Chr. 5, ST8SIA4).

An important intracellular factor, which has been described as a co-factor in dendrite growth, is dysbindin resp. dystrobrevin binding protein 1 (DTNPB1). It is particularly localized in axon bundles and especially in certain axon terminals^48^ and preserves the presynaptic calcium homeostasis as it is also involved in the transport of mitochondria to nerve terminals^49^. Thereby, it also has a functional role in synaptic vesicle biogenesis and neurite outgrowth^50,51^, as well as regulative function on dopaminergic and glutamatergic neurotransmission^52^. Genetic variants and reduced expression levels of DTNBP1 have significantly been linked to the pathogenesis of schizophrenia and bipolar disorder^50,51,53–55^.

Extracellular matrix proteins also play an important role in neuronal migration and the formation of dendrites^56^. Therefore, Reelin (RELN) was investigated in this study, which is, for example, secreted by Cajal–Retzius cells during cortical development. Besides migration in early development, RELN has also been shown to promote the maturation of dendrites and dendritic spines and modulate synaptic function in the later maturation status of the brain^57^. Alterations of its expression levels and secretion have been closely linked to the pathogenes of schizophrenia in the past^58,59^.

All in all, alterations of DNA methylation and/or expression in our comprehensive set of targets could give an impression of the impact of the different cannabinoids on synapses.

## Results

### Morphology and length of dendrites and branches

**Fig. 1:**
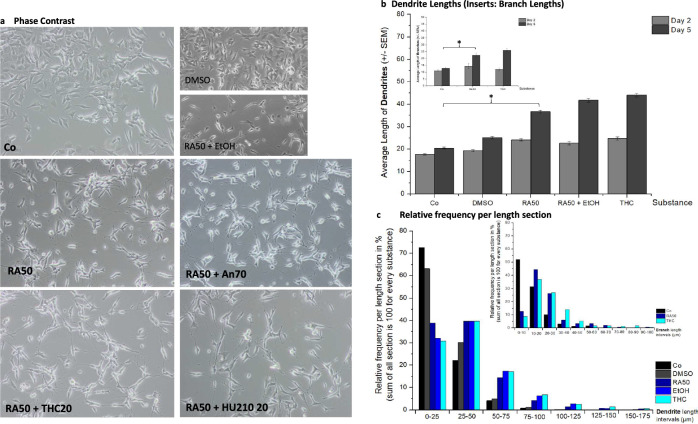
Morphology and dendrite lengths. **a** A representative phase contrast picture of every cell culture condition is depicted: undifferentiated control cells (Co), differentiated cells (treated with 50 µM retinoic acid, RA50), and differentiated cells additionally treated with 20 µM THC (THC20), anandamide 70 µM (An70), or HU210 20 µM (HU210 20). The smaller pictures show controls for the solvents of RA (DMSO) and THC (Ethanol, EtOH). **b** The average dendrite length of undifferentiated control cells (Co), differentiated cells (treated with 50 µM retinoic acid, RA50), and differentiated cells additionally treated with 20 µM THC (THC20) on day 2 and day 5. Additionally, controls for the solvents of RA (DMSO) and THC (Ethanol, EtOH) are shown. The smaller insert also indicates branch lengths for the three main conditions. Significant differences are indicated by *. **c** The presentation of the relative frequencies per length section (25 µm intervals) shows the distribution of different neurite lengths per condition. The smaller insert gives the same information for branches in 10 µm-intervals for the three main conditions.

As described earlier^9^, phase contrast microscopy revealed a pronounced morphologic difference between undifferentiated SH-SY5Y cells and 50 µM RA-treated cells (RA50) (Fig. 1a). Cells treated with RA50 built long dendrites and growth cones and showed dendritic branching whereas dendrites of untreated cells remained short. As DMSO was the solvent of retinoic acid and ethanol was necessary to dissolve THC, we also investigated whether these two solvents alone (in the case of DMSO) or in combination with RA (in the case of ethanol) could have an impact on morphology (smaller pictures in Fig. 1a). It becomes clear, that DMSO by itself did not affect morphology as application led to no alteration of morphologic features compared to untreated cells. The same was true for ethanol, as the application of ethanol (combined with RA50) showed no difference to cells solely treated with RA50.

Visually, application of the three tested cannabinoids Anandamide at a concentration of 70 µM (An70), 20 µM THC (THC20), and 20 µM HU-210 (HU210 20) led to a kind of blunting of neurites in the sense of less filigree growth cone structures with more or less big “knobs” at the end. Furthermore, there appeared to be more transport of vesicle-like structures along the neurites. As there were no obvious differences between the three cannabinoids, we focused on the most important cannabinoid in the ongoing discussion about the effects of cannabinoids on the brain: THC, when analyzing dendrite length and branching due to the great effort involved in that method.

Assessment of dendrite lengths at day 5 revealed comparable findings between cells solely treated with RA50 and those with additional application of THC, revealing an average length of 40 µm with an insignificant difference between means of both groups: 2.3 µm, *p* > 0.05 (Fig. 1b). Again DMSO and Ethanol (the latter in combination with RA 50) findings were added as controls to proof they had no effect by themselves. Like to be expected, untreated cells showed much shorter dendrites (in average 20 µm; comparison of controls with RA50 and THC20, respectively, revealed highly significant differences with *p*-levels <0.0001 in both cases). Substance effects were comparable for branches (inset in Fig. 1b, for a higher facility of inspection shown without solvent-controls), showing average branch lengths of about 25 µm for the sole application of RA50 and the combination with THC20 with a group difference of 4 µm (*p* = 0.1094). Branch lengths were significantly different between controls (approximately 10 µm) vs. RA50 and THC20, respectively, revealing group differences of 6.3 µm (*p* = 0.0001) and 10.3 µm (*p* < 0.0001). Solvents did not exert any effects.

On day 2 (beginning point of differentiation), extensions were short and similar under all conditions, with no significant differences between the groups.

The relative frequency of dendrites per length section is depicted in Fig. 1c. In the case of undifferentiated cells (Co, DMSO), the main portion (60–70%) of dendrites is to be found in the short section (0–25 µm) with decreasing portions in the following two sections (20–30% in 25–50 µm dendrites and 5% in 50–75 µm). From 75 to 100 µm on, there were almost no dendrites observed in control cells. Differentiated cells only showed a portion of 30–40% in the short section, about 40% in the section of 25–50 µm, still 20% of 50–75 µm long dendrites, and about 5% in the 75–100 µm section and there were also neurites with a length between 100 and 175 µm. For branches, the distribution patterns were comparable (inset, Fig. 1c, for better clarity without solvent controls).

### Mean methylation rates

**Fig. 2:**
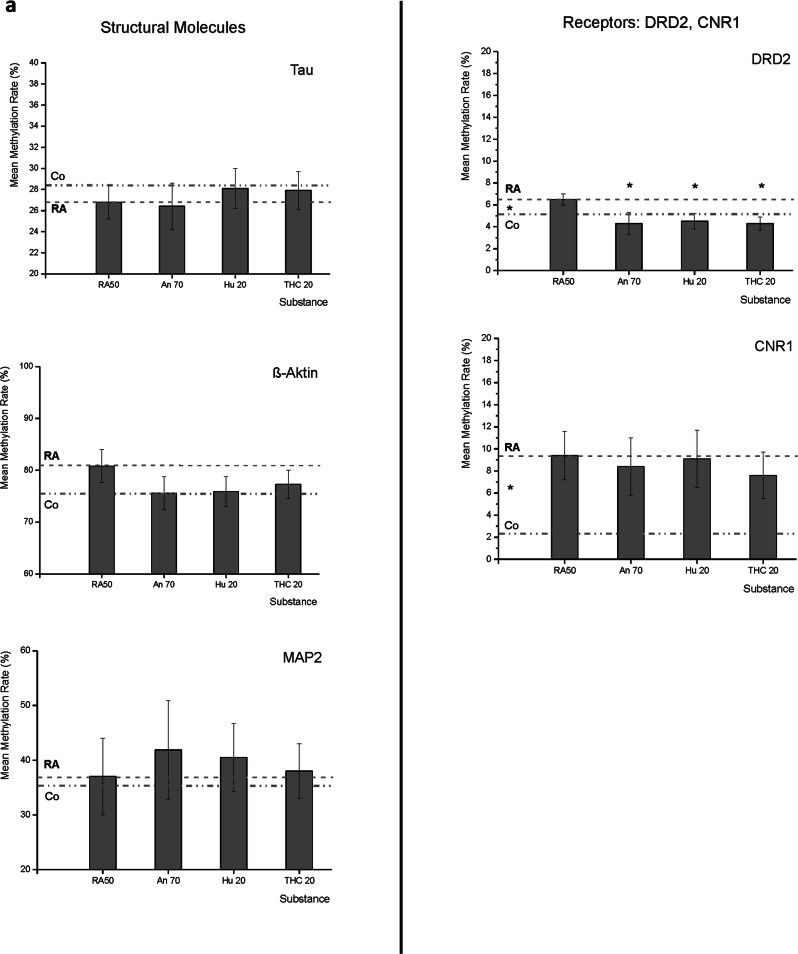

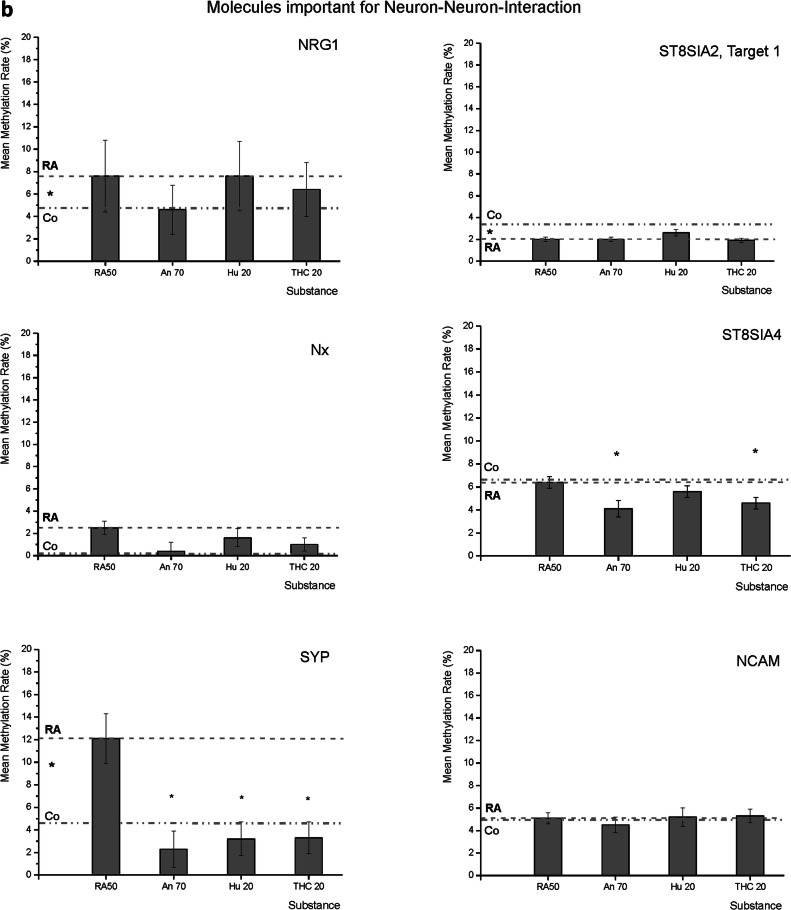
Mean DNA-methylation rates of target molecules. **a** Mean methylation rates of MAPT (Tau), ACTB (ß-actin), MAP2, DRD2, and CNR1 under the application of Retinoic Acid 50 µM (RA50) alone and in combination with either Anandamide (An70), HU210 (HU20) or tetrahydrocannabinol (THC20). For better subsumption of results, the broken line traces the RA50 level throughout the figure, and the dotted line represents the methylation rate of the respective target in undifferentiated control cells. Significant differences between controls and RA50-treated cells are indicated by * on the left between the two mentioned lines. In the case of DRD2, there were significant differences in methylation rates between differentiated cells and differentiated cells treated with Cannabinoids (indicated by * over the respective columns). In trend, cannabinoid treatment led to an approximation of methylation rates towards the level that was detected in untreated/undifferentiated control cells and also in the other investigated targets (besides MAP2). **b** Mean methylation rates of NRG1, NRXN1, SYP, ST8SIA2, ST8SIA4, and NCAM under application of retinoic acid 50 µM (RA50) alone and in combination with either Anandamide (An70), HU210 (HU20), or tetrahydrocannabinol (THC20). For better subsumption of results, the broken line traces the RA50 level throughout the figure, and the dotted line represents the methylation rate of the respective target in undifferentiated control cells. Significant differences between controls and RA50-treated cells are indicated by * on the left between the two mentioned lines. In the case of SYP, there were significant differences in methylation rates between differentiated cells and differentiated cells treated with Cannabinoids (indicated by * above the respective columns). In trend, cannabinoid treatment led to an approximation of methylation rates towards the level in untreated/undifferentiated control cells and also in the other investigated targets except for ST8SIA2 and 4.

We investigated the promoter region of eleven target molecules (the promoter region of one target was divided into two parts as the general methylation level was different in these two parts, distant from each other). Three of the investigated molecules are structural molecules (MAPT, β-Actin, and MAP2), two targets are receptors (DRD2, CNR1) (Fig. 2a, Suppl. Table 7), and the remaining six molecules are involved in neuron–neuron interaction (NRG1, NRXN1, SYP as well as ST8SIA2, ST8SIA4 and NCAM1) (Fig. 2b, Suppl. Table 7).

As the main focus of the present work is to investigate the effects of cannabinoids on differentiated/differentiating cells, the methylation values are given for the 50 µM RA (RA50) condition as well as for the three cannabinoids in the respective figures. Values for the undifferentiated cells are only indicated as a line in the figures in order to enable evaluation of the direction in which the values were developing under the application of cannabinoids in relation to the cells solely treated with RA50. The comparison between undifferentiated and differentiated cells was the subject of an earlier work^9^. To further facilitate inspection, we also inserted a line reinforcing the average methylation of RA50 across the whole x-axis. We indicated significant differences between controls and RA50 treated cells by an asterisk between the Control cells (Co) and RA50 line, left hand in the respective chart.

In most cases, the methylation status of retinoic acid-treated cells was higher than that of untreated control cells, with three exceptions: MAPT, ST8SIA2 target 1, and ST8SIA4.

Concerning the structural molecules, there were no significant differences between controls and RA50-treated cells. In the case of MAPT (Tau), the general methylation level was about 27%. In trend, controls showed slightly higher methylation, and the application of cannabinoids led to an approximation of differentiated cell methylation levels towards unmethylated controls in the case of HU210 20 and THC20. In β-Actin (ACTB) and MAP2, the general methylation level was higher than in MAPT (about 78% and 37%, respectively), and differentiated cells showed higher methylation than undifferentiated cells in trend, whereas there was almost no difference in MAP2. In the case of ACTB, the difference was more pronounced (5.5%) but still not significant. Under the application of cannabinoids, the values approached those of the undifferentiated controls.

Regarding methylation levels of the two investigated receptors, there were significant differences between the methylation status of controls and differentiated cells. The general methylation level was rather low (<10%) in both targets. In the case of DRD2, the methylation level was 6.5% (±0.5%) in differentiated cells, whereas it was 4.7% (±0.5%) in undifferentiated controls (*p* < 0.05). Interestingly, methylation levels were also significantly different from differentiated cells when cannabinoids were applied. Under all three cannabinoids, the methylation level fell even under the level seen in undifferentiated controls (An70: 4.3% ± 1%, *p* < 0.05; HU210 20: 4.5% ± 0.7%, *p* < 0.05; THC20: 4.4% ± 0.6%, *p* < 0.05). In CNR1, the methylation level in differentiated cells was 9.4% (±2.2%) and 2.2% (±3.7%) in undifferentiated cells, *p* < 0.05. The average methylation of differentiated cells was a bit lower under the application of cannabinoids, but the difference to cells solely treated with RA50 did not reach the level of significance.

In the group of molecules necessary for neuron–neuron-interaction, the general methylation was low (<15%), and retinoic acid-treated cells showed a higher methylation level than undifferentiated controls except in the case of the two enzymes concerned with sialylation of NCAM, namely ST8SIA2 (T1) and ST8SIA4.

NRG1 showed a methylation level of 7.6% (±3.2%) in the differentiation condition with RA50, whereas it was significantly lower in control cells with 4.6% (±4.2%), *p* < 0.05. In comparison to RA50, methylation levels decreased under the application of cannabinoids by trend (An70 4.6% ± 2%; Hu210 20 7.6% ± 3.1%; THC 20 6.4% ± 2.4%; *p* > 0.05). For NRXN1, we found a methylation level of 2.5% (±0.6%) in RA50-treated cells and 0% (±0.8%) in undifferentiated controls, *p* > 0.05. In trend, methylation levels were lower in case of additional treatment with cannabinoids approaching the level of undifferentiated controls (An70 0.4 ± 0.8; Hu210 20 1.6% ± 0.8%; THC 20 1% ± 0.6%; *p* > 0.05). Like in the case of NRG1, there was a significant difference between methylation levels of RA50 treated (12.1% ± 2.2%) and undifferentiated control cells (4.7% ± 2%), *p* < 0.05 in SYP. Furthermore, the application of cannabinoids led to a significant reduction of methylation levels in comparison to solely RA50-treated cells, even beyond the level of undifferentiated cells (An70 2.3% ± 1.6; Hu210 20 3.3% ± 1.5%; THC 20 3.3% ± 1.4%; all *p* < 0.05).

As mentioned before, control cells showed a higher methylation level than differentiated cells in the case of the two polysialic acid transferases. For ST8SIA2 (T1), there was even a significant difference (2% ± 0.2% vs. 2.7% ± 0.2%; *p* < 0.05). However, the application of cannabinoids led to almost no alterations in comparison to RA50-treated cells (An70 2% ± 0.2; Hu210 20 2.6% ± 0.3%; THC20 1.9% ± 0.2; *p* > 0.05). In ST8SIA4, methylation levels of RA50-treated cells and undifferentiated controls (Co) were almost identical (6.4% ± 0.5% vs. 6.5% ± 0.6%, *p* > 0.05), but interestingly, application of cannabinoids led to significantly reduced methylation levels in case of An70 and THC20: 4.1% ± 0.7%, *p* < 0.05 and 4.6% ± 0.5%, *p* < 0.05. Values for Hu210 20 were also lower but only by trend (5.6% ± 0.5%, *p* > 0.05). In the promoter of NCAM1, there were no noteworthy differences in methylation level between the different conditions (RA50 5.1% ± 0.5%; Co 5% ± 0.6%; An70 4.5% ± 0.7%; Hu210 20 5.2% ± 0.8%; THC20 5.3% ± 0.6%; *p* > 0.05).

### Expression: RNA-levels

**Fig. 3:**
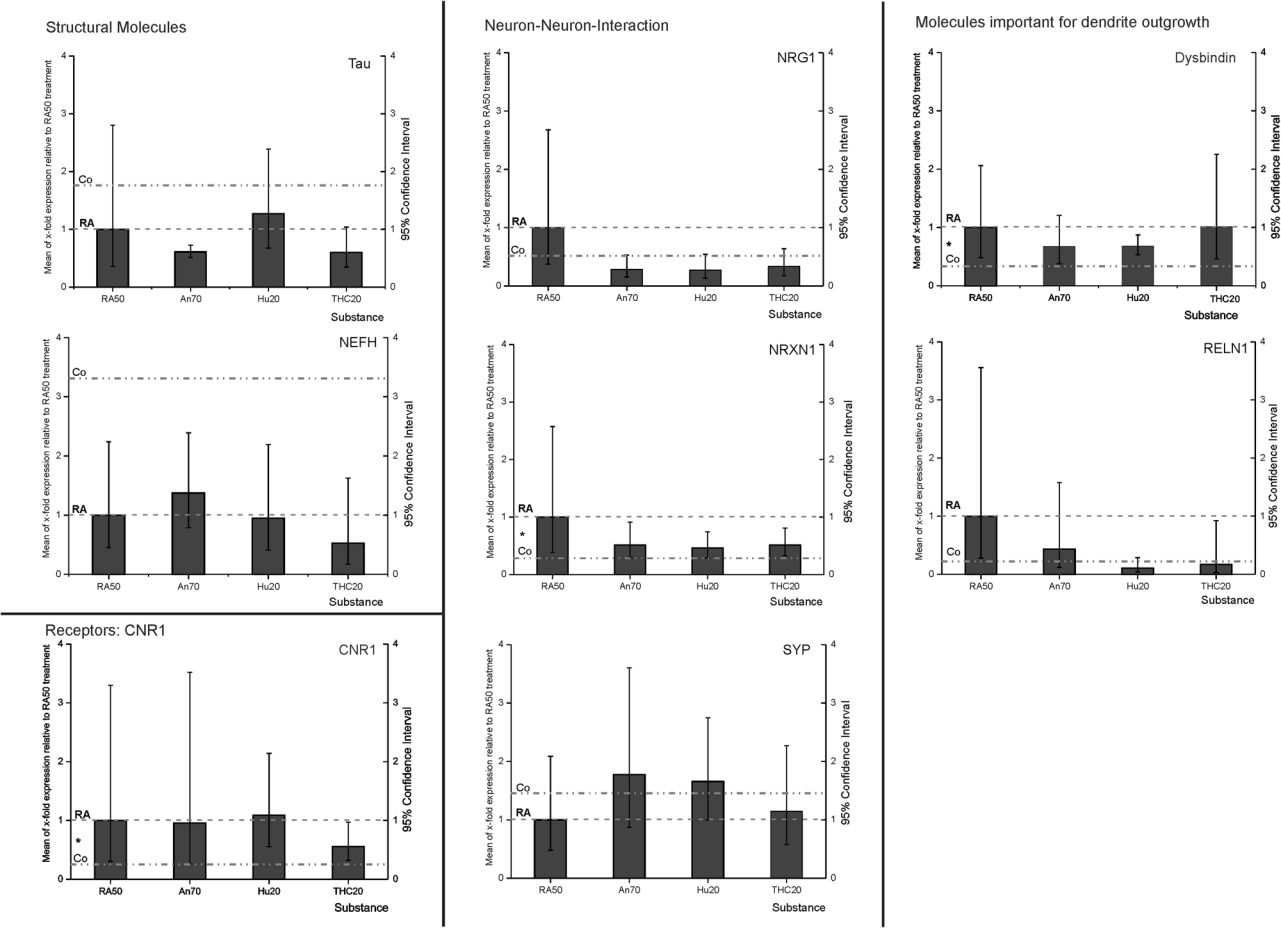
RT-qPCR RNA-expression data: mean *x*-fold expression (95% confidence interval) of MAPT (Tau), CNR1, NRG1, NRXN1, SYP, DTNBP, and RELN under application of retinoic acid 50 µM (RA50) either combined with anandamide (An70), HU210 (HU210 20) or tetrahydrocannabinol (THC20) in relation to expression in solely RA50-treated SH-SY5Y cells (=1). For better subsumption of results, the broken line traces the RA50 level throughout the figure and the dotted line represents the expression level of the respective target in undifferentiated control cells. Significant differences between controls and RA50-treated cells, as published before (Jahn et al.^9^), are indicated by * on the left between the mentioned lines. There were no significant differences in expression between differentiated cells and differentiated cells treated with Cannabinoids. However, in trend, cannabinoid treatment led to an approximation of expression towards the expression level in untreated/undifferentiated control cells in the investigated targets besides MAPT.

As mentioned in the methods part, it was not possible to investigate the RNA levels of all targets measured in the methylation analysis for technical reasons. Relative expression levels of all targets were related to RA50 (consequently, RA50 is always 1), and 95% confidence intervals are given in order to facilitate the evaluation of value distribution (Fig. 3, Suppl. Table 7).

Concerning the structural molecules, there were no significant differences between the different conditions at all. In the case of MAPT, relative expression levels were: RA50 1 (95% CI low 0.36, high 2.8), Co 1.63 (95% CI low 0.41, high 2.43), An70 0.6 (95% CI low 0.51, high 0.72), Hu210 20 1.27 (95% CI low 0.68, high 22.4), THC20 0.53 (95% CI low 0.35, high 1.04). NEFH showed the following expression levels relative to the RA condition: RA50 1 (95% CI low 0.45, high 2.24), Co 3.21 (95% CI low 0.33, high 3.5), An70 1.38 (95% CI low 0.8, high 2.39), Hu210 20 0.95 (95% CI low 0.41, high 2.19) and THC20 0.53 (95% CI low 0.17, high 1.63).

Regarding receptor CNR1, there was a significant difference in expression only between RA50 (1 (95% CI low 0.3, high 3.3) and Co (0.2 (95% CI low 0.1, high 1.6), *p* < 0.05. All other treatments did not induce a significant difference in relation to RA or to each other: An70 0.96 (95% CI low 0.26, high 3.52), Hu210 20 1.09 (95% CI low 0.55, high 2.15) and THC20 0.56 (95% CI low 0.32, high 0.97), although in the THC20 condition, there was an approximation towards undifferentiated control cells in trend.

The following three investigated targets belong to the group of neuron–neuron-interaction molecules. In NRG1, treatment with the three cannabinoids led to values even lower than in the control condition but findings did not reveal any significant differences in expression in relation to RA50: RA50 1 (95% CI low 0.37, high 2.7), Co 0.46 (95% CI low 0.3, high 1.84), An70 0.29 (95% CI low 0.15, high 0.53), Hu210 20 0.27 (95%CI low 0.18, high 0.64) and THC20 0.34 (95%CI low 0.18, high 0.64). In NRXN1, there was only a significant difference between RA50 (1 (95% CI low 0.39, high 2.58) and Co (0.31 (95% CI low 0.2, high 1.9)), *p* < 0.05. Values of all other treatments were approximating those of controls but were not significantly different from RA50 or from each other: An70 0.52 (95% CI low 0.29, high 0.91), Hu210 20 0.46 (95% CI low 0.29, high 0.74) and THC20 0.52 (95% CI low 0.33, high 0.81). Regarding SYP, expression levels in cells treated with the different cannabinoids showed an approximation to those of the control condition. However, there were no significant alterations: RA50 1 (95% CI low 0.48, high 2.09), Co 1.31 (95% CI low 0.38, high 2.64), An70 1.77 (95% CI low 0.87, high 3.6), Hu20 1.66 (95% CI low 1, high 2.5) and THC20 1.15 (95% CI low 0.58, high 2.27).

Regarding the two investigated molecules important for dendritic outgrowth, we could, in trend, also observe an approximation of the expression levels towards those seen in undifferentiated controls in cannabinoid-treated cells, although differences did not get significant in comparison to the RA50 condition. Analysis of DTNBP1 (Dysbindin) expression levels revealed a significant difference between RA50 (1 (95% CI low 0.49, high 2.06)) and Co (0.3 (95% CI low 0.24, high 4.17)), *p* < 0.05. Under treatment with cannabinoids, values were in between RA50 and Co but did not show significant differences toward RA50 or each other: An70 0.68 (95% CI low 0.38, high 1.21), Hu210 20 0.68 (95% CI low 0.53, high 0.88), THC20 1 (95% CI low 0.46, high 2.26). For RELN1, there were no significant differences at all. RA50 1 (95% CI low 0.28, high 3.6), Co 0.24 (95% CI low 0.2, high 3.72), An70 0.44 (95% CI low 0.12, high 1.6), Hu210 20 0.11 (95% CI low 0.04, high 2.9) and THC20 0.17 (95% CI low 0.03, high 0.92).

### General remarks

As we saw no significant differences between the three cannabinoids in the analyses of methylation rates or expression levels by real-time qPCR, we decided to focus on delta-9-THC in the remaining expression analyses (ICC and western blot), like we also reduced data points from morphology to determination of dendrite lengths in the earlier section.

In order to figure out if we might have missed short-term changes in RNA-expression, we also performed RNA-analyses on days 2–4 but could also not detect any significant expression differences (data not shown).

### Expression and localization: ICC

**Fig. 4:**
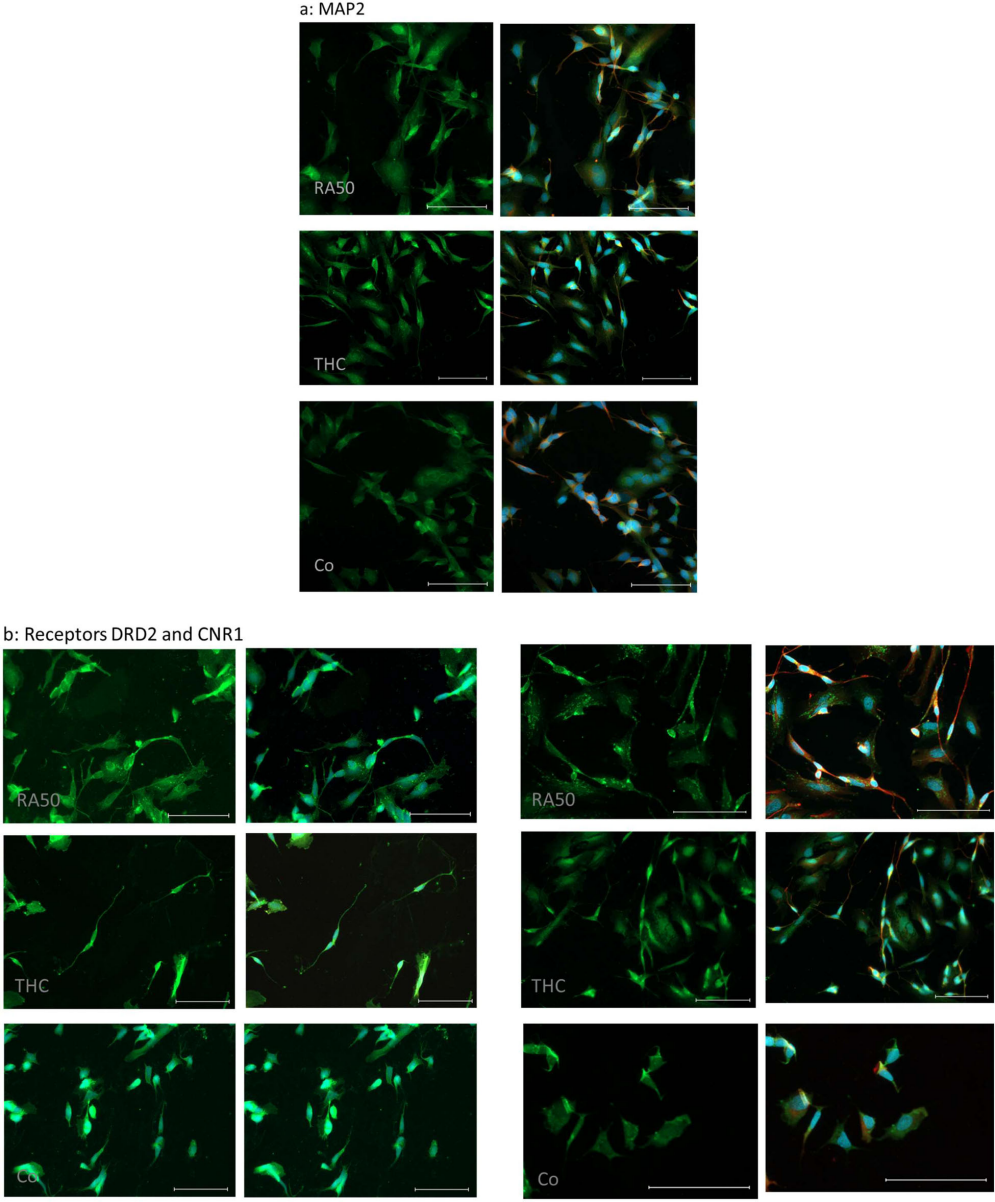

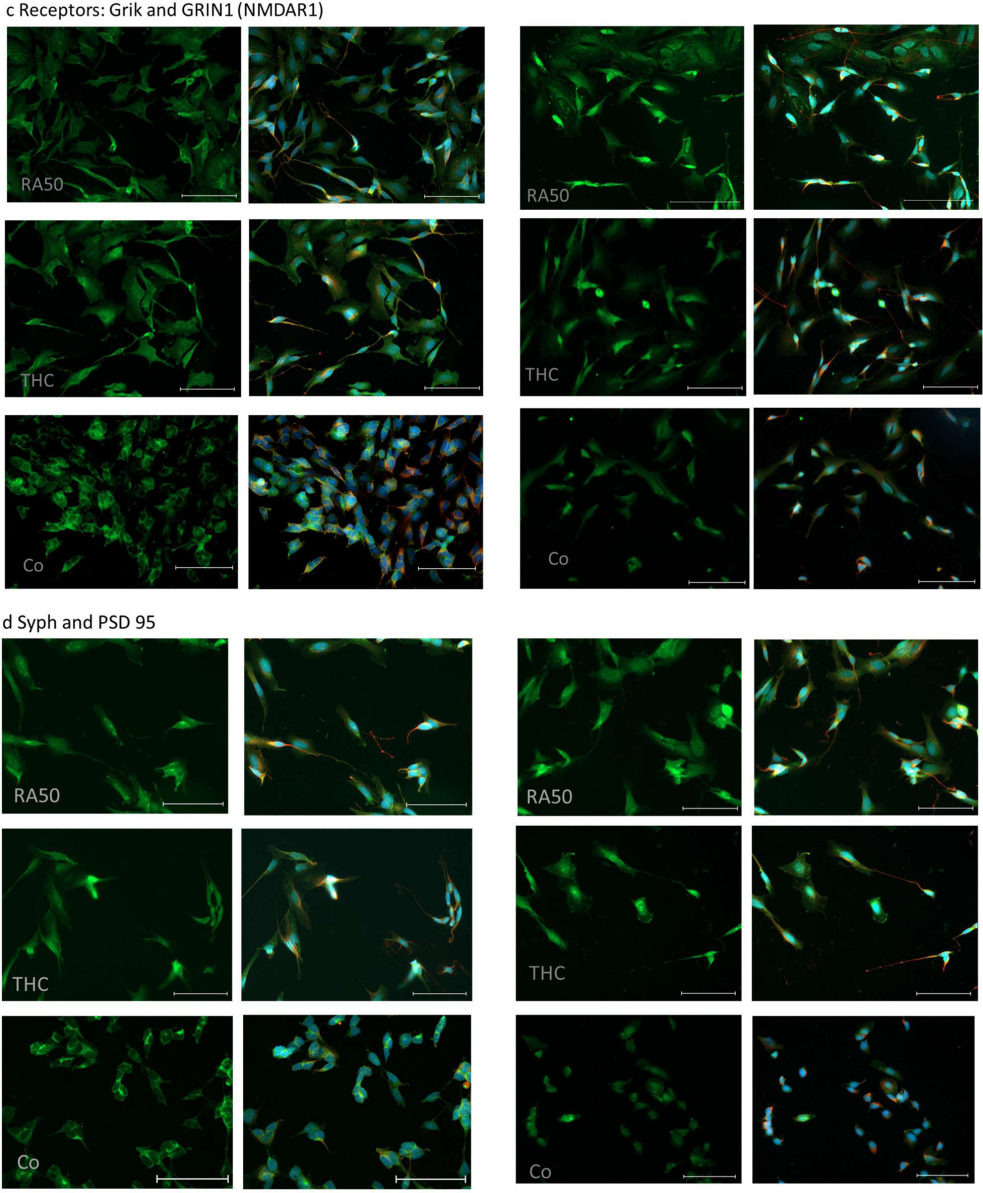
ICC of target molecules. **a** ICC of MAP2 in differentiated cells, THC-treated cells, and undifferentiated control cells. On the left side, sole MAP2-staining (green) is presented, whereas, on the right side, the corresponding merge pictures of the co-staining with TUBB3 (βIII-Tubulin) (red) and nuclei (blue) are depicted. The scale bar represents 100 µm. **b** ICC of DRD2 (left panel) and CNR1 (right panel) in differentiated cells, THC-treated cells and undifferentiated control cells. In the respective left column sole DRD2- and CNR1-, staining (green) is presented, whereas, in the right column, the corresponding merge pictures of the co-staining with TUBB3 (βIII-Tubulin) (red) and nuclei (blue) are depicted. Unfortunately, in the case of the DRD2 staining TUBB3 has not been stained for practical reasons. Scale bar: 100 µm. **c** ICC of Grik (left panel) and GRIN1 (NMDAR1) (right panel) in differentiated cells, THC-treated cells, and undifferentiated control cells. In the respective left column sole Grik- and GRIN-, respectively, staining (green) is presented, whereas, in the right column, the corresponding merge pictures of the co-staining with TUBB3 (βIII-Tubulin) (red) and nuclei (blue) is depicted. The scale bar represents 100 µm. **d** ICC of SYP (left panel) and DLG4 (PSD95) (right panel) in differentiated cells, THC-treated cells, and undifferentiated control cells. In the respective left column, sole SYP- and DLG4-staining (green) is presented, whereas, in the right column, the corresponding merge pictures of the co-staining with βIII-Tubulin (red) and nuclei (blue) is depicted. The Scale bar represents 100 µm.

In line with our earlier study^9^, the fluorescence intensity of MAP2 was higher in differentiated (RA50) cells than in controls. Furthermore, the distribution patterns were slightly different. While it was rather homogenous in controls, distribution showed a dot-wise pattern in RA50-treated cells. Between THC20 and RA50, there were no obvious differences, neither concerning fluorescence intensity nor the distribution pattern (Fig. 4a).

Regarding the four investigated receptors, we saw, in most cases, an approximation of findings in THC20-treated cells toward controls. In DRD2, CNR1 (Fig. 4b), and NMDRA1 (Fig. 4c, right panel), the distribution pattern in RA50 cells was dot-wise, whereas it was rather homogenous in control cells. THC20-treated cells also showed a more homogeneous distribution pattern. In the case of GRIN1 (NMDAR1), control cells additionally showed a lower fluorescence intensity. Grik (Fig. 4c, left panel) showed a homogeneous distribution in both, control and RA50 cells, but it was detectable in neurites exclusively in RA50 cells. In the case of Grik, THC cells did not show special differences in comparison to cells solely treated with RA50.

SYP (Fig. 4d, left panel) showed a dot-/or cluster-wise distribution in controls and RA50-treated cells. THC20 cells showed a homogenous distribution of SYP.

ICC of DLG4 (PSD95) (Fig. 4d, right panel) showed a less intense fluorescence in undifferentiated control cells compared to RA50 and THC20-treated cells. The few signals in control cells were located in a dot-wise manner, whereas signals were more homogeneously distributed in RA50 cells. In THC20-treated cells, we found more dot-wise signals again, also in those cells with a clear neuron-like cell shape.

### Expression: Western Blots

**Fig. 5:**
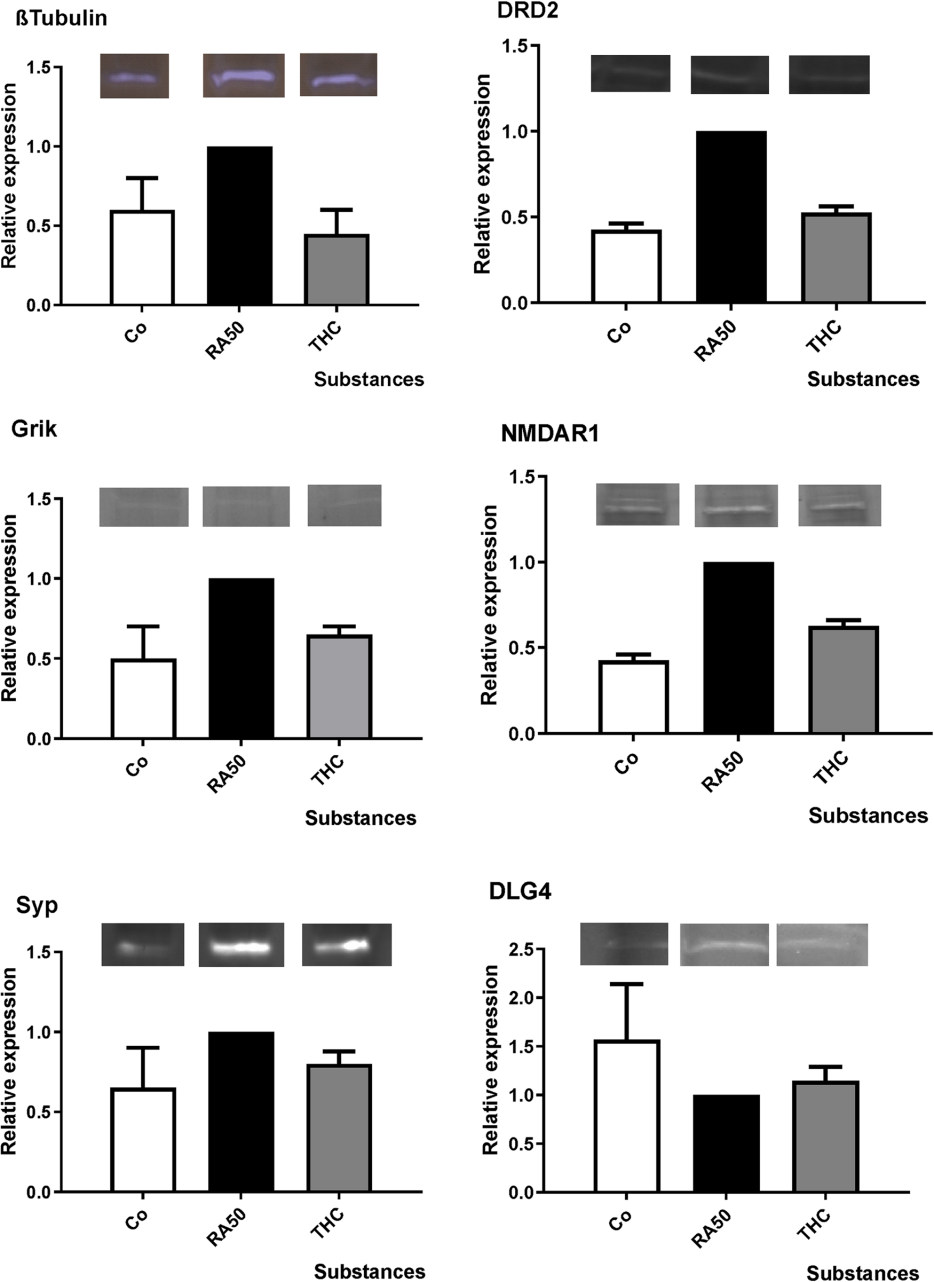
Western blot analysis: mean x-fold expression (±SD) of TUBB3 (βIII-Tubulin), DRD2, Grik, GRIN (NMDAR1), SYP and DLG4 (PSD95) in undifferentiated control cells, under application of 50 µM retinoic acid (RA50) in combination with THC20 in relation to expression level in solely RA50-treated SH-SY5Y cells (=1). There were no significant differences in expression between differentiated cells and differentiated cells that were additionally treated with Cannabinoids. However, in trend, cannabinoid treatment led to an approximation of expression toward the expression level in untreated/undifferentiated control cells in the investigated targets. Representative bands are shown over each column for each target and condition. N-numbers of western blot experiments for the different targets were: TUBB3: 3, DRD2: 2, Grik: 2, NMDAR: 2, SYP: 2, DLG4: 3.

Western Blot analyses of the six investigated targets did not reveal any significant group differences (*p* > 0.1 in all cases), but expression levels showed the same trend for undifferentiated controls and THC-treated differentiated cells (figure). In TUBB3 (βIII-Tubulin), DRD2, Grik, GRIN (NMDAR1), and SYP, we observed a lower expression of the respective targets in control and THC-treated cells in comparison to RA-differentiated cells. In the case of DLG4 (PSD95), the protein expression was higher than in RA-treated cells (Fig. 5, Suppl. Table 7).

## Discussion

Until today, there is a kind of controversy about whether cannabinoids exert positive or negative effects on the brain in the context of schizophrenic psychoses. It has even been speculated that consumption of THC could be interpreted as a kind of self-medication in schizophrenic patients^60^. Cannabidiol has even been tested as an adjunctive therapy option in schizophrenia^61^.

Undoubtedly, cannabinoids can, in general, exert a wide range of supportive effects on the human body, such as pain relief^62^. There are even hints for neuroprotection in neurological diseases like stroke^63^ and Parkinson´s disease^64^, for example. However, in contrast, most studies imply that the use of cannabinoids can have rather detrimental effects, for example, on cognition like fragmented thinking, disturbances in short-term memory, and others^65^. Besides these temporary disturbances, it has been observed that the onset of schizophrenic psychosis was closely related to preceding THC consumption^66,67^. Probably, supportive as well as adverse effects of cannabinoids might be a question of the individual developmental stage and age as well as the current health condition (physiologic vs. pathologic stage), either restoring or destroying the natural homeostasis in the endocannabinoid system.

In recent years, there has been growing evidence for a key role of the endocannabinoid system during neuronal development. The endocannabinoid system mainly comprises CNR1 in the brain but also, to a lesser extent, CNR2 (cannabinoid receptor type 2), which is else more prominent in the periphery. During early ontogenetic development, CNR1s are deeply involved in neurogenesis, glial differentiation, neural migration, and elongation of axons^12,68^. Also later, the endocannabinoid system still has a great impact on adolescent-specific ontogenetic processes like the development of the dopaminergic system in the prefrontal cortex, for instance^69^. In the pathogenesis of schizophrenia, puberty/early adulthood is a critical time point as the first episode of psychosis often occurs in this stage of life^70^. The “dual-hit model” assumes that some individuals have a certain susceptibility for the later development of schizophrenia, which might be caused by a “first hit” like inborn subtle and symptomless changes in the brain. Later on, an environmental trigger like trauma, cannabis consumption, or other stressors (the “second hit”) can then aggravate the situation, especially during puberty when extensive hormonal changes take place, leading to the onset of psychosis^71^. It is well known that there is a physiological loss of synapses of about 30% during puberty, which is much higher in schizophrenic patients (about 60%)^5^.

We were therefore interested in whether synapses can be lastingly affected by external cues like THC via an alteration of DNA-methylation patterns in genes important for synapse formation and preservation. Alterations of methylation patterns are a well-known link between environmental cues and a stable alteration of expression patterns without affecting the DNA sequence itself^72^. There are already some hints that cannabinoids like Cannabidiol can alter DNA methyltransferase (DNMT)-activity^10^. Accordingly, we investigated the influence of cannabinoids on DNA-methylation patterns as well as the respective expression levels of synapse molecules (besides the morphology of cells) in our SH-SY5Y cell culture model for neuronal differentiation^9^.

Although we could observe subtle morphologic differences between differentiated SH-SY5Y cells and differentiated cells treated with the three cannabinoids, like more structures resembling transport vesicles and kind of blunting of growth cones (Fig. 1a), there were no significant differences with regard to neurite or branch lengths (Fig. 1b, c). During the last few years, there has been increasing evidence that the endocannabinoid system is highly involved in the regulation of neuronal shape and structure^73^, which is mainly maintained by filamentous Actin and stable microtubule networks. Findings are controversial as under activation of CNR1, some authors found neurite retraction, whereas others showed neurite outgrowth^74^. The cytoskeleton is mainly regulated by Rho-like GTPases, which have a high impact on actin polymerization, focal adhesion, tubule dynamics, and membrane transport^75^. Our finding of a kind of blunting of growth cones is in line with the fact that activation of CNR1 by Anandamide can lead to a contraction of the neuronal actomyosin-cytoskeleton, most probable by coupling of G_12_/G_13_ proteins that mediate ROCK-mediated non-muscle myosin II (NM II) activation, which is reversible but can be more stable under chronic CNR1 activation^75^. Under artificial conditions such as overexpression of CNR1, Anandamide induced a ROCK-dependent rounding of cells^76^. Berguis et al. observed a RhoA- and ROCK-dependent repulsion of growth cones under CNR1-activation^77^. Our observation of increased numbers of structures resembling transport vesicles could hint toward an increased transport of CNR1-molecules of treated cells as it has been shown that modulation of growth cone dynamics and axonal pathfinding by the endocannabinoid-system (Anandamide-induced CNR1-activation) during development strongly depends on the delivery and precise presentation of the respective receptors at the growth cone surface^78^. However, it could also point to a compensatory process as Xu et al. could show a significant reduction of CNR1 under consumption of cannabis in a positron emission tomography (PET)-study^79^ and, as already mentioned, we observed a blunting of the growth cones under application of cannabinoids.

Methylation rates were investigated in the promoter region of MAPT (Tau), ACTB (βIII-Actin), MAP2, DRD2, CNR1, NRG1, NRXN1, SYP, NCAM, ST8SIA2 and ST8SIA4 (Table 1, Fig. 2a, b). In general, methylation rates were higher in differentiated cells (RA50) as compared to undifferentiated controls, at least in trend, except for MAPT and both ST8SIAs (2 and 4) showing higher methylation in controls (whereas in the latter target, ST8SIA4, there was almost no difference between both groups). Differences reached the level of significance in the case of DRD2, CNR1, NRG, SYP, and ST8SIA2. In general, higher methylation in differentiated cells could appear to be kind of surprising, as it is usually assumed that higher methylation leads to lower expression and vice versa^80^. However, it has been shown that correlations do change during development^81^. Furthermore, higher methylation rates are often found in more mature cells^82^, as in higher differentiation, most genes become less accessible. Surprisingly, methylation levels of control and differentiated cells pointed in the opposite direction compared to our previous differentiation paper in the case of CNR1, NRG1, and SYP^9^ (in the case of DRD2, there were no significant differences before, and ST8SIA2 was not part of the previous study). However, it is possible that cells, depending on the passage they have been in before starting the experiment, need different time spans to react towards cues like differentiating agents and/or to reach the point of final differentiation. Therefore, it is conceivable that the affected genes still had to remain readable for a certain time span before reaching final differentiation in our earlier experiments as compared to the current study. Nevertheless, concerning the focus of our current study (with the same and comparable ground conditions in all groups), it was eye-catching that the application of cannabinoids led to an approximation of respective values towards those of the undifferentiated controls in the great majority of our investigated targets, even though differences of cannabinoid treated cells did only become significantly different from differentiated cells in some cases. As already mentioned before, it is known that Cannabidiol (as another important phyto-cannabinoid) can regulate the DNMT (DNA-methyltransferase) activity either by directly interacting with the enzyme or indirectly via neurotransmitter-mediated signaling^10^. The impact of delta-9-THC on DNA methylation has mainly been investigated with regard to the germ line. It has been shown that THC consumption can lead to altered methylation patterns, especially in autism-associated genes in sperm-DNA^83^. Furthermore, there are hints that prenatal exposure to delta-9-THC can also lead to changes in DNA methylation in genes concerned with neurobehavioral development^84^. Regarding the targets for which we found significant differences (either only between controls and differentiated cells or also in the comparison between differentiated cells and those additionally treated with cannabinoids), it is striking that these molecules belong mainly to the groups of receptors (DRD2, CNR1) and the molecules that are important for neuron-neuron interaction (NRG1, SYP, and ST8SIA2).

**Table 1:** Target names, corresponding gene symbols, and chromosomes, as well as information on which methods targets could be investigated (for technical reasons, not all methods were applicable for all targets).

Target	Gene symbol/chromosome no.	Methylation rate-data available	Expression data available, RNA: RNA expression WB: western blot/protein expression ICC: immunocytochemistry
Structural molecules			
Microtubule-associated protein Tau	MAPT/ 17	Yes	Yes: RNA
Actin beta	ACTB/7	Yes	
Microtubule-associated protein 2	MAP2/ 2	Yes	Yes: ICC
Neurofilament-heavy polypeptide	NEFH/22		Yes: RNA
ßIII- tubulin	TUBB 3		Yes: WB, ICC
Receptors			
Dopamine receptor 2	DRD2/ 11	Yes	Yes: WB, ICC
Cannabinoid receptor 1	CNR1/ 6	Yes	Yes: RNA, ICC
Ionotropic glutamate receptor 5, sensitive to kainate	Grik5/19		Yes: WB,ICC
Ionotropic glutamate receptor, sensitive to glutamate and synthetic N-methyl-d-aspartate	GRIN1/9		Yes: WB, ICC
Molecules important for neuron–neuron-interaction			
Neuregulin-1	NRG1/ 8	Yes	Yes: RNA
Neurexin	NRXN1/ 2	Yes	Yes: RNA
Synaptophysin	SYP/ X	Yes	Yes: RNA, WB, ICC
Postsynaptic density	DLG4/17		Yes: WB, ICC
Dysbindin1 (dystrobrevin binding protein1)	DTNBP1/6		Yes: RNA
Reelin	RELN/7		Yes: RNA
Polysialic acid transferase 2 ST8 alpha-N-acetyl-neuraminide alpha-2,8-sialyltransferase	ST8SIA 2/5	Yes	
Polysialic acid transferase 4 ST8 alpha-N-acetyl-neuraminide alpha-2,8-sialyltransferase 4	ST8SIA 4/5	Yes	
Neural cell adhesion molecule	NCAM1/ 11	Yes	

Highlighted with a grey background are those targets of which we have methylation data AND expression data (of any kind, either *RNA*-, protein-expression, or immunocytochemistry).

As already mentioned in the introductory part, DRD2 is of special importance with regard to the pathogenesis of schizophrenia. Therefore, our finding of a significant rapprochement of the methylation status toward undifferentiated controls under the application of cannabinoids could give a hint toward a significant aversive effect of cannabinoids on the dopaminergic system. In CNR1 as part of the endocannabinoid system, there were also significant differences between methylation patterns of undifferentiated cells vs. differentiated ones, but cannabinoids only lead to a trend-wise approximation of values towards undifferentiated controls. Even though the level of significance was just missed, our findings could point to a disturbance of the sensitive homeostasis in the endocannabinoid system during differentiation under the artificial application of cannabinoids.

NRG1, as an essential molecule for synapse formation, was significantly different between undifferentiated controls and differentiated cells. Application of cannabinoids led to only trend-wise rapprochement of values towards undifferentiated controls, unobtrusively pointing to an aversive impact of cannabinoids on synapse formation. SYP, as an important indicator of synaptic vesicle turnover, showed a significant approximation of values towards undifferentiated controls under the application of cannabinoids, indicating a disadvantageous alteration of synaptic transmission by cannabinoids.

ST8SIA2 (alpha-2,8-sialyltransferase 8b isoform 1/2 precursor (Chr. 15)) is an enzyme like ST8SIA4 (cmp-n-acetylneuraminate-poly-alpha-2,8-sialtransferase (Chr. 5)), which both sialysate NCAM. Sialysation of NCAM hinders homo- and heterophilic binding of NCAM, leading to a reduction of cell-cell contacts and neurite outgrowth^44^. In contrast to the other targets, which also showed significant differences, ST8SIA2 methylation was higher in control cells. Nevertheless, under the application of cannabinoids, there was no difference with regard to differentiated cells, not even in trend. Therefore, one could conclude that cannabinoids probably do not interact with NCAM sialysation, at least not via alterations of the DNA-methylation status.

Remarkably, there were no differences in effects between the different cannabinoids we investigated, belonging to the three classes of cannabinoids: endogenous (Anandamide), exogenous (phytocannabinoids, THC), and synthetic (HU-210) cannabinoids, at least not in the concentrations we used. Even HU-210 showed an equalization of values towards undifferentiated cells, although it has been shown to be neuroprotective in retinal degeneration^85^. However, neuroprotective properties have been more ascribed to its enantiomer HU-211^16^, and we did not use a stress model in our study. This seems to support the hypothesis that the direction of the effect of cannabinoids seems to depend on the ground state of cells. Nevertheless, it might be argued that it could also be a matter of concentration that we used in our study. Respective concentrations for our experiments have been determined in preliminary tests as it is difficult to conclude from serum drug levels (of schizophrenic patients consuming THC, for example) on the extracellular concentration in the brain. To our knowledge, no respective microdialysis studies exist in the human brain. Apart from that, our experiments were conducted in vitro, and often, concentrations have to be adapted from those determined in vivo. Furthermore, although the affinity in terms of inhibitory constants (Ki) of the cannabinoids we used here are widely known from ligand-binding assays (THC- Ki: 10 nM^14^, Anandamide-Ki: 58,3 nM^15^, HU-210 Ki: 0,06 nM^15,86^) it is hard to predict how effective they are as they are metabolized differently in the cell. Furthermore, THC is only a partial agonist, meaning that it does not entirely induce the intracellular signal cascades associated with the CB1 receptor. Therefore, it can also act as a partial antagonist^87^ by competing with the, at least in vivo, physiologically abundant Anandamide. Another factor complicating any predictions is that there are not only receptor-mediated effects of cannabinoids^88^. Therefore, we validated the effect and toxicity of different concentrations of the respective cannabinoids in preceding experiments (representative phase contrast pictures can be found in Supplemental Fig. 1A to 1C for all tested concentrations of substances). Even though some other authors used lower concentrations in the existing literature, i.e., 10 µM THC^89^, 10 µM HU-210^90^, and 10 µM Anandamide^91^, we identified higher optimal concentrations: 20 µM THC, 20 µM HU-210, and even 70 µM Anandamide. In our study, these were the concentrations that turned out to already have subtle effects on the morphology of the cells with regard to neurite and growth cone structure^77^ but did not yet induce cell death. Differences in concentration levels of cannabinoids can result from different study conditions like the setting (toxicity assays^89^ vs. others), the time substances were applied (for example, in one study only for 48 h^92^), and other factors. A noteworthiness about our approach is, for example, that we used RA in a concentration of 50 µM for the neuronal differentiation of the SH-SY5Y cells, as in-depth discussed in an earlier study^9^. As this is a stronger differentiation cue than 10 µM of RA, it is conceivable that also higher concentrations of cannabinoids are necessary to impact the system. Besides the earlier described advantage of the application of 50 µM RA over the application of 10 µM on neuronal differentiation parameters like inhibition of proliferation, neurite length, branching, and formation of growth cones^9^, it might also lead to a faster differentiation. Usually, in most cell culture conditions (cell lines, primary cells, and so on), some of the cells do not react towards treatment, depending on the cell cycle stage they are in at the beginning of the treatment, and can remain in the original status. In SH-SY5Y cells, this means that some of the cells maintain their tumor characteristics and keep proliferating. Therefore, when keeping the cells for a longer time period in culture, the proportion of proliferating/undifferentiated cells can increase and eventually predominate from a certain time point. Therefore, to keep the differentiated cells the pre-dominating ones, it is preferable to reach a fast differentiation and to keep the observation period as short as possible. Especially, the control condition without any application of RA, otherwise tends to over-proliferation. It might be argued that 5 days is not a long observation period. However, for our purpose, it was efficient as morphological features were clearly visible. Furthermore, the aversive effects of cannabinoids on the human brain become mostly visible in developmental stages. Therefore, we were more interested in the effects of cannabinoids on developing synapses and applied the cannabinoids from the stage of seeding/beginning of differentiation, resulting in an observation period of 5 days for the effects of the cannabinoids, whereas other studies first had to differentiate the cells with resulting shorter observation period of 48 h for the substances of interest afterward^92^.

So far, our findings could be interpreted in terms of the disadvantageous effects of cannabinoids on neuronal connections and synapses. As discussed in the following part, accordingly, also expression data confirmed this remarkable “value-equalization effect” with undifferentiated controls under the application of cannabinoids.

The expression levels of MAPT (Tau), NEFH, CNR1, NRG1, NRXN1, SYP, DTNBP1, and reelin gene 1 (RELN1) have been investigated on RNA-base by RT-qPCR (Table 1, Fig. 3). We observed a significantly higher expression of CNR1 (receptor endocannabinoid-system), NRXN1 (neuron–neuron-interaction) and DTNBP1 (neurite outgrowth, calcium homeostasis) in differentiated cells. In trend, there was also a higher expression level of NRG1 (neuron-neuron-interaction) and RELN1 (extracellular matrix protein) in differentiated cells. The structural molecules (MAPT and NEFH), as well as SYP (neural transmission), did not show any significant differences between differentiated and undifferentiated cells. In trend, MAPT and NEFH seemed to be more expressed in controls, whereas there was only a negligible difference between both groups in SYP. Application of cannabinoids led to an approximation of expression levels towards undifferentiated control cells except for the structural molecules, in which the expression level remained on the level of differentiated cells or even below that. The fact that we missed significance in cannabinoid application might be due to moderate *n*-numbers of preparations that qualified for the final analyses (5–6) or maybe due to short-term alterations of the expression level. However, to roughly control for the latter possibility, we also measured expression levels on days 2–4 but did not find any significant expression peaks (data not shown). It is conceivable that RNA expression peaks last much shorter than would be detectable in a 24-h interval (so-called “minute-changes”^93^) and were, therefore, not identified with our approach.

The expression levels for the structural molecule TUBB3 (βIII-Tubulin), receptors DRD2, Grik, and GRIN1 (NMDAR1), as well as for the synapse-function related targets SYP and DLG4 (PSD95) (Table 1, Fig. 5) were determined on protein base. Except for DLG4, measurements revealed a higher expression of these targets in differentiated cells as compared to undifferentiated control cells, albeit only in trend. Under the application of delta-9-THC, values assimilated to the level of undifferentiated cells (Fig. 5). Although the level of significance was not reached, the overall picture seems to be very consistently pointing to an expectably higher presence of the target proteins in differentiated neuron-like cells. In this case, the reduction of values could be interpreted as a de-differentiating effect of cannabinoids on our neuron-like cells. Target proteins were not only quantitatively measured but also visualized by ICC (Fig. 4) in order to get an impression of their localization and distribution pattern in the cell. TUBB3 has been used as a co-staining in every target ICC to facilitate localization. As the cell line we used in our cell-culture model (SH-SY5Y) is a neuroblastoma cell line, undifferentiated cells also express already TUBB3^9^. Additionally, also MAP2 and CNR1 have been stained. In general, fluorescence intensity was higher in RA50-treated cells in MAP2 and DLG4, although insignificant western-blot data revealed the impression of somewhat higher DLG4 expression in the controls. Intensity vanished again under treatment with cannabinoids. As MAP2 is strongly involved in the formation of dendrites and DLG4 is important for the clustering of receptors, the higher fluorescence intensity in differentiated cells seems to be conclusive, and reduction in intensity under application of cannabinoids might, therefore, point to a loss of these neuronal properties. Localization pattern showed a more dot-wise distribution, especially of the receptors DRD2, CNR1, and GRIN (NMDAR1) in RA50-treated cells in comparison to a homogenous distribution in undifferentiated controls and THC-treated cells. Considering the requirements in neurons, it seems to be comprehensible that receptors are concentrated at the surface where neurites from other cells approach in order to form synapses, leading to a dot-wise arrangement. In contrast, the undifferentiated neuroblastoma cells might offer receptors in a more widespread manner in preparation for a potential approach of a neurite in order to facilitate aggregation wherever needed^9^. The fact that cannabinoids lead again to a more homogenous distribution pattern strongly points to a loss of synaptic connection as also indicated by the loss of fluorescence intensity of DLG4. For Grik 1, the distribution pattern was homogenous in all conditions. In neurites, however, it was only visible in differentiated cells and even with THC application. SYP showed a punctate distribution in control and differentiated cells. In control cells, these dots were concentrated in the area of the inner cell body, whereas in THC-treated cells, the distribution became more homogeneous. Furthermore, this molecule was visible in neurites only in the RA50 condition and, to a lesser extent, in the THC condition. Staining of the latter two target molecules in the area of dendrites might point to the transport of the proteins towards the axon boutons^22^. In the case of Grik, this transport seems not to be influenced by cannabinoids that much, whereas it was attenuated in the case of SYP. Fittingly, Hu et al. found reduced levels of axon bouton marker SYP in schizophrenia^22^. Grik, as a very specific (non-NMDA/Kainate) glutamatergic receptor, is strongly regulated by RNA editing and RNA splicing^94^ and might, therefore, be more resistant towards an impact of cannabinoids, which are known to have an influence on DNMTs rather^10^. However, unfortunately, we do not have any methylation data or more quantitative expression data on Grik. Nevertheless, all in all, most targets investigated in our current study show a clear development toward the methylation values, expression levels, and distribution pattern of undifferentiated control cells under the application of cannabinoids.

## Conclusions

To sum up, our study results point to a “de-differentiating” effect of cannabinoids rather than a beneficial impact on neuron-like cells and their synaptic connections. Although the level of significance has not been reached in every case, the findings are consistent across all investigated target molecules and methods and, therefore, strongly indicate a disadvantageous effect of cannabinoids on neuronal networks in terms of a loss of neuronal properties, at least in developing neuronal networks like in our study.

### Limitations

For technical reasons, it was not possible to investigate all targets with all methods. However, expression data were available for all targets either from real-time or western-blot analysis. Higher *n*-numbers would have been desirable in some cases as the level of significance was probably missed in some cases due to low *n*-numbers, at least in the case of RNA- and protein expression data. However, our n-number of measurement repetitions is in the normal range, which is normally seen in most respective cell culture studies, depending on the method. We had 10 preparations, all of which could be used for dendrite length analysis. For technical reasons, there were some drop-outs for methylation analysis (depending on the target: 7–9 preparations worked out), in real-time measurements (5–6 per target were available), and western blots (duplicates or triplicates were reliably analyzable). It might be argued that the SH-SY5Y cell culture model is, in general, a very simple model. However, for the analyses of synapses on a very molecular level, it is of great advantage as more complex systems have many unknown parameters that could indirectly interfere with the actual object of investigation, which makes it almost impossible to investigate direct effects. Furthermore, SH-SY5Y cells are of human origin. And, last but not least, it is always desirable to have alternatives to animal experiments for ethical reasons.

## Material and methods

### Nomenclature of target molecules

In the current study, many synapse markers were investigated by different methods comprising investigation of gene modifications (methylation analysis, real-time quantitative polymerase chain reaction RT (RT-qPCR)) but also protein expression and localization (western blot, Immunocytochemistry (ICC)). Due to the high number of investigated targets, it might have been confusing to use gene symbols in some parts of the paper and the corresponding protein terms as abbreviations in other parts. Therefore, target molecules are continuously indicated by their gene symbols (Table 1) in every part of the manuscript. For technical reasons, it was not possible to investigate each of the 18 targets with every method Table 1 also gives an overview of which target could be investigated with which method.

### Cells

SH-SY5Y cells, a thrice-cloned cell subline of the neuroblastoma cell line SK-N-SH, were bought from ATCC® (American Type Culture Collection, CRL-2266). Thawing and progeny were performed according to the manufacturer’s protocol.

### Cell culture medium

The basic medium was composed of DMEM (Dulbecco’s Modified Eagle Medium)/Ham’s F12 (1:1) with stable glutamine (Biochrom, FG 4815), supplemented with 10% fetal calf serum (FCS) superior (Biochrom, S0615) and 1% penicillin–streptomycin (P/S; PAN, P06-07100). This basic medium was used purely as a negative control condition.

### Cell proliferation

After thawing, cells were proliferated in T25 cell culture flasks (Sarstedt®). When reaching about 80% confluence, cells were transferred to T75 cell culture flasks (Sarstedt®). Splitting was performed by washing cells with phosphate-buffered saline (PBS; Biochrom, L182-01) once and incubating them with 2.5 ml trypsin-ethylenediaminetetraacetic acid (EDTA, PAA, L11-004) for 5 minutes at 37 °C. To stop the digestion, 5 ml medium was added, and the whole mix was centrifuged for 5 min at 140*g*. The supernatant was discarded, and the cell pellet was resuspended in a medium and replated into a T75 cell culture flask or used for further experiments. The maximum amount of these passages was 10.

### Media adjuncts

Retinoic acid (RA) (Sigma-Aldrich, R2625) for neuronal-like differentiation of cells has to be resolved with dimethylsulfoxide (DMSO; Sigma-Aldrich, 41640). Therefore, another negative control condition was the basic medium in addition to 0.1% DMSO.

Concerning the cannabinoids used in the current study, the respective concentrations for the experiments have been determined in preliminary tests (also compare the discussion part on applied concentrations). For all substances, those concentrations were finally chosen, which turned out to already exert subtle effects on the morphology of the cells but did not yet induce cell death (Suppl. Fig. 1A–C).

Anandamide (Tocris, 1339) as an endogenous CNR1 agonist was finally applied in a concentration of 70 µM. It is delivered as a 14.39 mM solution in ethanol (5 mg/ml ethanol). In the text, it is referred to as An70.

Hu210 (HU is the abbreviation for the Hebrew University, where it was originally developed by Prof. R. Mechoulam) as a synthetic CNR1 agonist was obtained from Sigma (H7909) and had to be resolved in DMSO. It was finally applied at a concentration of 20 µM. Throughout the text, it is indicated by Hu210 20.

For the usage of Delta-9-THC (Sigma, T2386), official permission from the German “BfArM -Bundesinstitut für Arzneimittel und Medizinprodukte/ Bundesopiumstelle” was needed. The permission number was 458 38 08. Delta-9-THC was obtained as a 79.5 mM solution in ethanol (5 mg/ml ethanol) and was used in a final concentration of 20 µM in the cell culture experiments. In the text, we use the abbreviation THC20 for this condition.

### Coating of glass coverslips

Laminin (from Engelbrecht-Holm Swarm murine sarcoma basement membrane; 1 mg/ml, Sigma-Aldrich, L2020) was used in order to mimic the extracellular matrix in the brain. After thawing on ice (to avoid aggregation), the Laminin solution was diluted 1:50 (20 µl per ml pure DMEM). After 3 h, coated coverslips were washed with PBS before cell seeding.

### Cell seeding, duration of the treatment, and the number of preparations

For differentiation, 100,000 cells were seeded on coated glass coverslips of 12 mm diameter in 24-well Cell Culture Plates (Cellstar, Greiner Bio-One®) per well. For every substance/condition, cells were incubated for 5 days (also according to our previously established protocol^9^), and the medium, including fresh addition of the respective cannabinoids, was exchanged on day 3. Four wells were utilized per condition and preparation. Every condition (control, 50 µM Retinoic Acid (RA50), Anandamide, Hu210, THC, DMSO, Ethanol) was tested in 10 independent cell preparations. For technical reasons, n-numbers in results can vary, depending on the method: All could be used for dendrite length analysis. For technical reasons, there were some drop-outs for methylation analysis (depending on the target: 7–9 preparations worked out), in real-time measurements (5–6 per target were available), and western blots (duplicates or triplicates were reliably analyzable).

### Analysis of morphology and cell counting

The morphology of cells was analyzed using an Olympus Microscope (type CKX41) with an Olympus camera (XC 30) and the imaging software Cell^D^. Photos were taken along the “equator” of a well on days 3 and 5 after seeding. For determination of the number of cells as well as lengths of the dendrites, cell bodies were counted, and dendrites were precisely measured in pictures of 10 different preparations with the help of the “Inkscape” software (Fig. 1). Corresponding statistics were done with GraphPad Prism 10 by the use of an unpaired *t*-test.

### DNA- and RNA-extraction/sample preparation

DNA and RNA extraction was done by means of the Allprep Kit (Qiagen®). Extracted DNA was stored at −20 °C, and RNA at −80 °C in case it could not be directly transcribed into complementary DNA (cDNA).

### RNA transcription into cDNA

For transcription of RNA into cDNA for further downstream analysis, the iScript Kit (Bio-Rad®, 170-8890) was used. In total, 500 ng of RNA was applied in addition to 1 µl transcriptase and 4 µl iScript reaction Mix. For a final reaction volume of 20 µl, nuclease-free water was added. In a CFX 96 cycler (Bio-Rad®), the following cycling protocol was used for transcription: 25 °C for 5 min., 42 °C for 30 min, 85 °C for 5 min, and 4 °C for 1 h. If not used immediately after transcription, cDNA was stored at −20 °C.

### Methylation analysis

After DNA extraction, the individual DNA concentration of each sample was measured by means of the Nanodrop (Peqlab®), and 500 ng per sample was bisulfited and purified by use of the EpiTect Kit (QAGEN, Hilden, Germany, 59104). In order to amplify the sequences of interest (semi-) nested PCRs were performed by use of the HotStarTaq Master Mix Kit (Qiagen, 203445). According to the manufacturer’s protocol, the reaction mix was composed of the following: 1 µl bisulfite-converted DNA (or 1 µl of PCR1 product in case of preparing a second PCR), 5 µl HotStarTaq Master Mix Kit, 0.4 µl primer forward (20 pmol/µl), 0.4 µl primer reverse (20 pmol/µl) and 3.2 µl nuclease-free water. Primer sequences, the position of analyzed targets, as well as the number of analyzed CpGs and PCR temperatures, are provided in Supplemental Table 1. The Sequencing PCR and Sequencing, according to Sanger, were performed by means of the BigDye Terminator v3.1 Cycle Sequencing Kit (Applied Biosystems, 4337455). The reaction mix for the sequencing PCR was composed of 30 ng (max. 6.9 µl) PCR-product, 0.5 µl Big Dye 3.1, 2.0 µl Big Dye Buffer, 0.6 µl primer forward or reverse (5 pmol/µl) and x µl nuclease-free water to obtain a total volume of 10 µl (BigDye® Terminator v3.1 Sequencing Kit (Applied Biosystems, Foster City, CA, USA, 4337455)). Parameters for the (semi-) nested touchdown-PCR were chosen like following: 95 °C for 15 min (min), 97 °C for 2 min, followed by 15 cycles of 95 °C for 30 s, X °C (temperature depending on target, see Supplementary Table 3) for 45 s and 68 °C for 1 min, followed by 15 cycles of 95 °C for 30 s, X–15 °C for 90 s and 68 °C for 2 min. Samples were then kept at 68 °C for 4 min and afterward at 12 °C until they were removed from the cycler. Sequencing PCR protocol was by default 96 °C for 60 s, followed by 28 cycles of 96 °C for 10 s, 50 °C for 5 s and 60 °C for 4 min. Finally, samples were kept at 12 °C in the cycler until further processing. The automatized purification of products from the (semi-) nested touchdown-PCR was performed by use of Agencourt®AMPure®XP beads (Beckman Coulter GmbH, Krefeld, Germany, A63881) on a Biomek MC96 (Beckman Coulter GmbH, Krefeld, Germany). For the clean-up of sequencing PCR products, Agencourt®CleanSeq® (Beckman Coulter GmbH, Krefeld, Germany, A29154) was used on a Biomek MC96 (Beckman Coulter GmbH, Krefeld, Germany). Sequencing was performed on an Applied Biosystems ® 3500 xL DNA Analyser (Applied Biosystems, Foster City, CA, USA) in 15 µl HIDI (Formamide, Applied Biosystems, 4311320).

### Methylation data analysis

Sequence trace files were first processed by the ESME software package in order to determine the DNA methylation rate. “ESME” automatically normalizes signals, corrects for incomplete bisulfite conversion, and executes a quality control^95^. Exclusively CpG-sites showing valid results in >70% of all samples are then used for further analysis. Base positions are labeled as negative in promoter regions and positive from the beginning of the first exon, according to the common consensus, while the first base of the first exon corresponds to position zero. Statistical analysis of methylation rates was performed by usage of IBM SPSS Statistics 26 (IBM, New York, NY). The linear mixed model was used to analyze differences in DNA methylation between different cell culture conditions. Mean methylation rates of the whole region analyzed were compared between different cell culture conditions. Data are given as mean ± S.E.M. (Fig. 2a, b and Suppl. Table 7A). Only differences with *p*-values < 0.05 were considered significant.

### Expression analysis

#### Real-time PCR

Real-time PCRs were performed by use of the Go Taq Master Mix Kit (Promega®, A6002), based on SYBR green. The reaction mix contains 12.5 µl SYBR green, 0.5 µl primer forward, 0.5 µl primer reverse, 1 µl cDNA, and 10.5 µl nuclease-free water to obtain a total volume of 25 µl. In general, the position of primers was chosen in a way that they depicted the most important transcripts. At least one primer of each respective primer pair covered an exon-exon border in order to prevent binding on potential DNA contamination (Supplemental Table 2). The “NetPrimer” software and BLAST (Basic Local Alignment Search Tool)-database were used to check the primers for secondary structures and target specificity. The reference genes (Supplemental Table 2, RG) used in the current study were chosen in accordance with a publication by Hellemans et al., who found them to be the most stable ones in neuroblastoma cells^96^. The following cycler program was used on a CFX96 cycler (Bio-Rad®): 95 °C for 2 min., followed by 45 cycles of 95 °C for 30 s, Y °C (depending on the target, see Supplemental Table 3) for 30 s, 72 °C for 30 s. Finally, samples were run at 68 °C for 1 min. The melt curve was achieved by a stepwise increase of 0.5 °C (8 sec.) to a final temperature of 95 °C. In the end, 16 °C was applied for 10 min. All samples were measured in triplicates. Besides ideal PCR temperatures (Y °C), primer efficiencies, length of amplicon, and concerned chromosome are to be found in Supplemental Table 3.

### Analysis of real-time qPCR data

Data were analyzed by use of the qBase software (Biogazelle®, Belgium), performing multi-reference gene normalization, geNorm, and data quality control according to the minimum information for publication of quantitative real-time PCR experiments (MIQE) guidelines^97^. Real-time expression data are given as relative quantities (ratio of expression in solely differentiated (RA-treated cells) in relation to expression in differentiated cells additionally treated with one of the cannabinoids and respective 95% confidence intervals (Fig. 3, Suppl. Table 7B). Only group differences with *p* < 0.05 were considered significant (Mann–Whitney *U* test).

### Immunocytochemistry

Cells were fixed with 4% paraformaldehyde (Merck, 4005) for 20 min, then washed with PBS (Invitrogen, 14190-094) and pre-incubated for 30 min with 5% bovine serum albumin (BSA, Sigma-Aldrich, A-9647) in PBS/0.3%Triton-X-100 (Sigma-Aldrich, T8787). The first primary antibody was applied in 1% BSA in PBS/0.3%Triton-X-100 for 90 min, followed by a PBS-washing step. The first secondary antibody was added in 1% BSA in PBS for 60 min. Afterward, cells were washed with PBS. In the case of double staining, an intermediate blocking step was performed by application of 5% BSA in PBS for 30 min. The second primary antibody was used in 1% BSA in PBS for 90 min. After washing with PBS, the second secondary antibody was applied in 1% BSA in PBS for 60 min. Mostly, cells were additionally stained with 4′,6-Diamidin-2-Phenylindol (DAPI) nuclear staining (Chemicon, S7113) in PBS (dilution 1:1000) for 10 min. All steps were performed at room temperature. After staining, cells were either fixed with 80% Ethanol (Ethanol absolute, HPLC, Baker, 8462; diluted with distilled water) in 24 well plates or they were mounted (Fluoroshield Histology Mounting Medium, Sigma-Aldrich, F6182) on object slides and additionally sealed with nail polish. An overview of secondary and primary antibodies that were used to target proteins specific for neurons and essential for neurite and synapse formation with respective dilution rates is shown in Supplemental Tables 4 and 5. An additional Supplemental Figure (Suppl. Fig. 2) depicts a representative figure of an ICC negative control for the Alexa 488 secondary antibody. Exemplary pictures for all kinds of staining are shown in Fig. 4.

### Western Blots

For Western Blots, a fluorescence detection approach was used. SH-SY5Y cells were lysed in 1× Laemmli Buffer (Bio-Rad #1610737), containing a 1× protease inhibitor cocktail (PI, Cell signaling #5871S). After adding 2-Mercaptoethanol (Sigma-Aldrich #60-24-2), the samples were denatured at 95 °C for 5 min, and stored at −80 °C until further processing. For analysis, the samples were loaded on gels made by use of the TGX Stain-Free^™^ Fast-Cast^™^ Acrylamide Kit (Bio-Rad #1610181) or on Mini-Protean TGX (ready-to-use) Gels (Bio-Rad, #4561094). After electrophoresis (100–120 V), the resolved proteins were transferred to an ethanol-activated Mini-size low-fluorescence polyvinylidene fluoride (LF PVDF) membrane (Bio-Rad #10026934) with a Trans-Blot^®^Turbo^™^ Transfer System at 1.3 mA, 25 V for 7 min or 1.3 mA, 25 V for 10 min for high molecular weight proteins. The PVDF membrane was blocked with 5% milk powder (Carl Roth #T145.2) in 1× tris-buffered saline (TBS) at RT for one hour, followed by incubation with the respective primary antibodies (Supplemental Table 6) at 4 °C overnight. After incubation with 1:500 diluted goat anti-rabbit secondary antibody StarBright^™^ Blue 700 (Bio-Rad #12004162) at RT for one hour, the fluorescent signal was detected with ChemiDoc MP Imaging System (Bio-Rad) (measurement wavelength of 660–720 nm). Normalization was done to total protein. Total protein bands were visualized with UV-excited stain-free TGX Gel using the Chemidoc MP Imaging System (BioRad®). After blotting, total proteins were measured by using the program-setting “stain-free blot.” The ImageLab 6 software (Bio-Rad) was used for normalization (to total protein) and relative quantification in relation to cells solely treated with 50 µM RA (therefore, there are no standard deviations/SEM for the RA50, which is consecutively always 1). Experiments were run in duplicates or triplicates depending on the target (Fig. 5, Suppl. Table 7C). Statistics were done with GraphPad Prism via a one-way ANOVA.

## Supplementary information

Supplementals

## Data Availability

Data are available from the corresponding author upon request via Jahn.Kirsten@mh-hannover.de.
