# Supplementary material for: Impact of cannabinoids on synapse markers in an SH-SY5Y cell culture model
Source: Schizophrenia (Heidelb). 2024 Oct 25;10(1):96. doi: 10.1038/s41537-024-00498-6 (PMC11502758; doi:10.1038/s41537-024-00498-6)
Supplement: Supplementary file 1 — Supplementals [file 41537_2024_498_MOESM1_ESM.pdf]

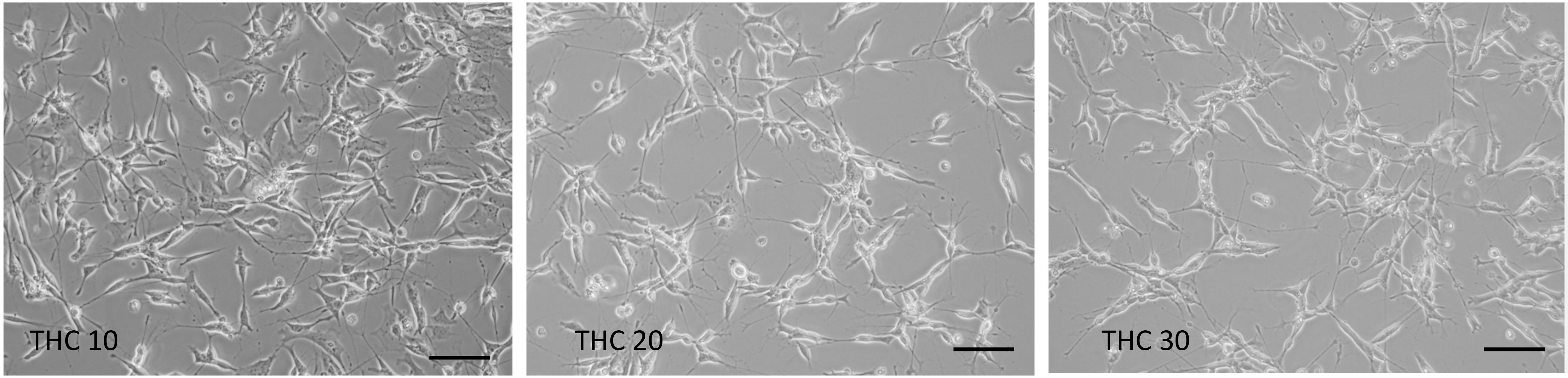

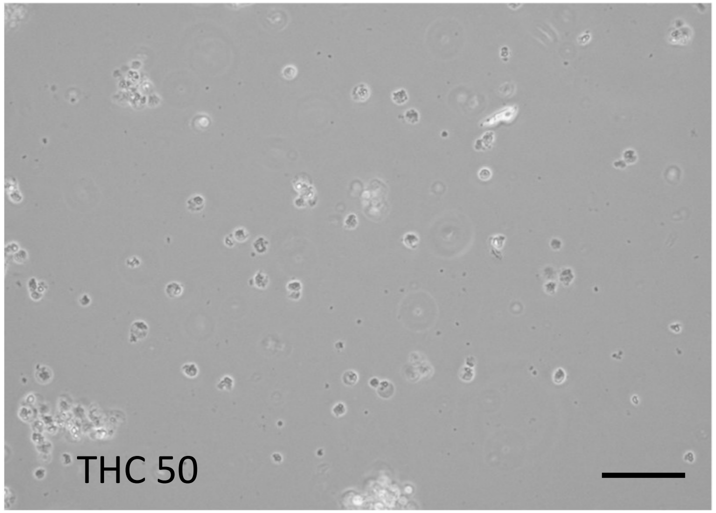

THC 10: 10 µM THC, only a few transport vesicle-like structures
THC 20: 20 µM THC, a lot of vesicle-like structures
THC 30: 30 µM THC, only subtle differences towards THC 20 (with cells eventually showing more cell detritus)
THC 50: 50 µM THC, strong apoptotic effects (rounding and detachment of cells)

Suppl. Figure 1a: phase contrast pictures of differentiated cells under additional application of 20 µM THC compared to other tested concentrations during preliminary experiments that were performed to figure out the most suitable concentration for our purpose. THC at a concentration of 20 µM has been chosen as it was the lowest concentration that clearly already affected neurites but did not show toxic effects. Scale bar: 50 µm.

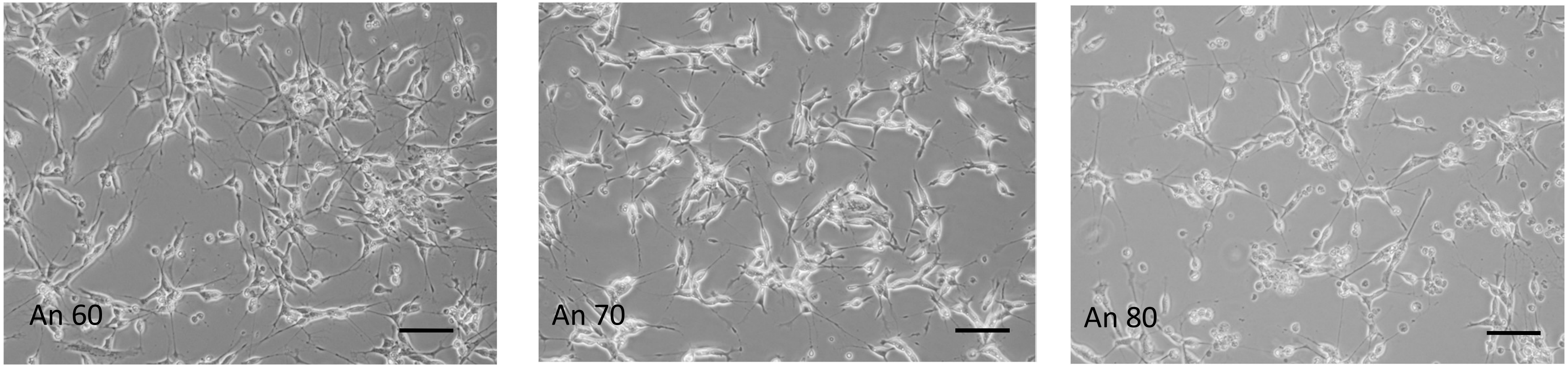

An 60: 60 µM Anandamide, only few transport vesicle- like structures, no blunting of neurites.
An 70: 70 µM Anandamide, a lot of vesicle-like structures, blunting of neurites
An 80: 80 µM Anandamide, first toxic effects appear (rounding and detachment of cells)
An 90: 90 µM Anandamide, strong apoptotic effect.

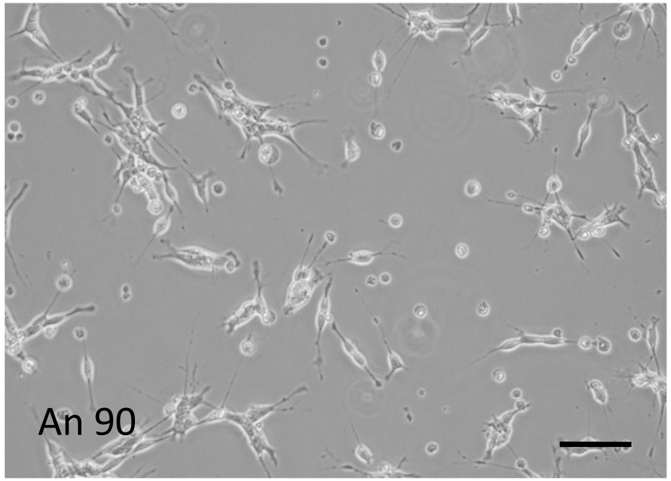

Suppl. Figure 1b: phase contrast pictures of differentiated cells under additional application of 70 µM Anandamide in comparison to other tested concentrations during preliminary experiments that were performed to figure out the most suitable concentration for our purpose. Anandamide at a concentration of 70 µM has been chosen as it was the lowest concentration that clearly already affected neurites but did not show toxic effects. Scale bar: 50 µm.

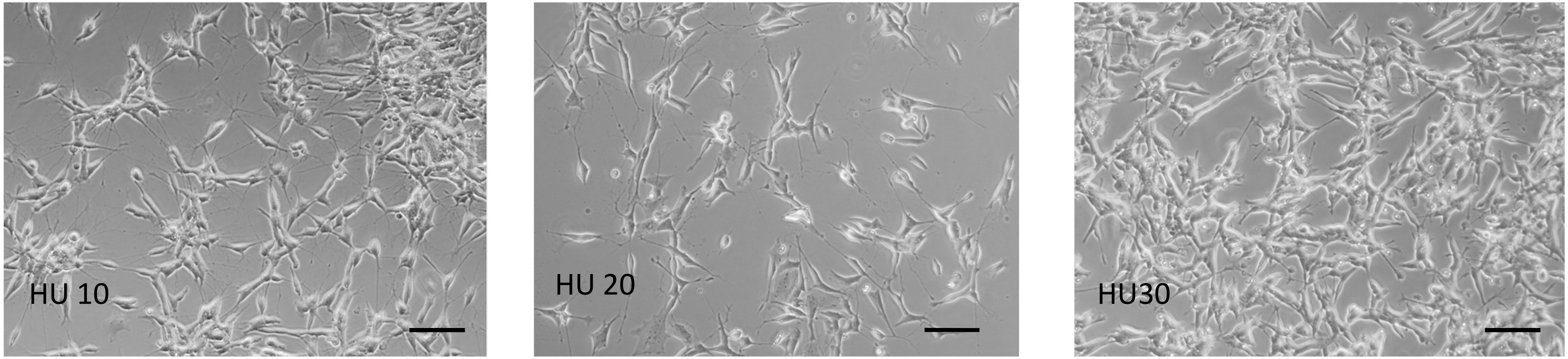

HU 10: 10 µM HU210, only a few transport vesicle-like structures, no blunting of neurites.
HU 20: 20 µM HU210, a lot of vesicle-like structures, blunting of neurites
HU 30: 30 µM HU210, first toxic effects appear (cell detritus appear)
HU 40: 40 µM HU210, strong apoptotic effect.

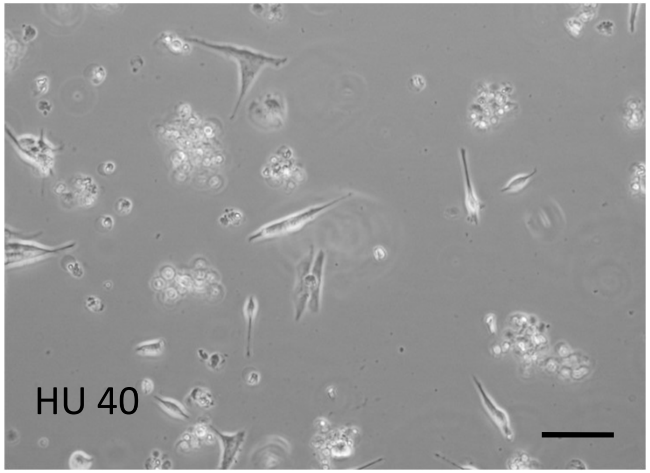

Suppl. Figure 1c: phase contrast pictures of differentiated cells under additional application of 20 µM HU210 in comparison to other tested concentrations during preliminary experiments that were performed to figure out the most suitable concentration for our purpose. HU210 at a concentration of 20 µM has been chosen as it was the lowest concentration that clearly already affected neurites but did not show toxic effects. Scale bar: 50 µm.

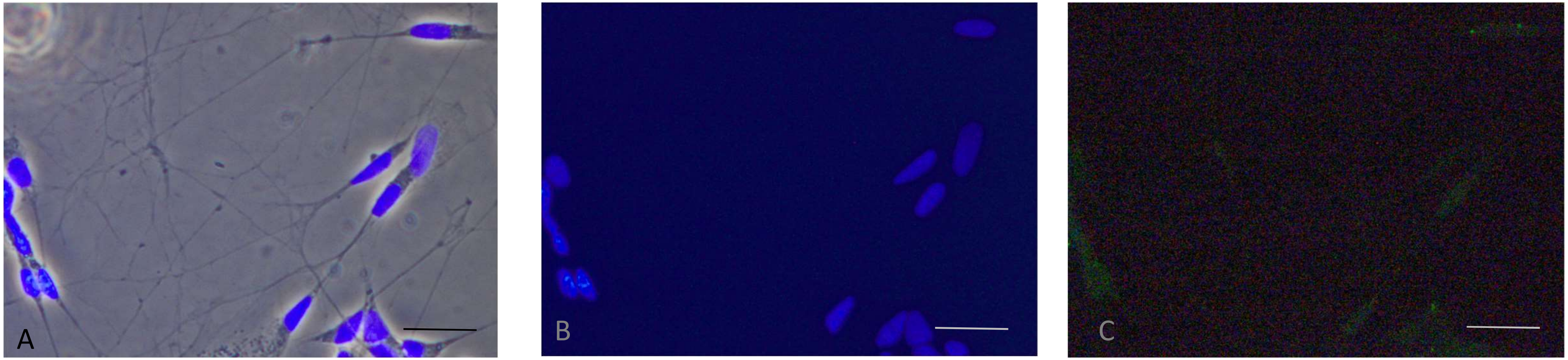

Suppl. Figure 2: Exemplary picture of a negative control for the A488 secondary antibody.
A: Merge of a transmission light picture and DAPI nuclear staining, B: pure DAPI nuclear staining, C: pure A488 secondary antibody
Scale Bar: 50 µm.

Gene	Gene Symbol/ Chromosome	Primers	CpGs were mapped between base positions:	Number of CpGs analysed in this region:	PCR Temperature X (°C)
Tau (Microtubule Associated Protein Tau)	MAPT/ 17	Fw_o: TAATAAAAAAGGTGGGAAAAAA,<br>Fw_i: AAAGGAAGTAGTTTGGGG,<br>**Rev: AACCTCCCCAAAAAAAAAAA**	-337 to +27	49	70
ß-Actin	ACTB/ 7	Fw: GTTTAGGTTGGAGTGTAGTG<br>**Rev_i: TTCACTCTTATTACCCAAACT**<br>Rev_o: CCAAACTAAATAAAACTAAACTC	-601 to -59	30	70
Microtubule Associated Protein 2	MAP2/ 2	Fw_o: TTTTGTTTTTTTTGGATGTATTTGTGGTT,<br>Fw_i:ATGTATTTGTGGTTGGGTTTGTGA,<br>Rev: TACTACTACTACTACTACTACCCA,<br>**Rev_Seq: AACCTTCTCCAAAACCCTAAT**	-484 to -297	12	70
Dopamine Receptor 2	DRD2/ 11	Fw_o: GTAATTTTTGGTTTTTGAGTT,<br>Fw_i:GAGGAGGTATAGTTTTTTTGGT,<br>**Rev_i: CTACTTAAAACTTCCAACTCCC**,<br>Rev_o:CACAAACTTCTAATCCTAACCT,	+132 to +461	53	62
Canabinoid Receptor 1	CNR1/ 6	Fw_o: GTATGTATTAGTTTTTTTTTGGTTT,<br>Fw_i: TTGGTTTGGTTAGGGTGTGA,<br>**Rev: CTATCTCTAACTCCCTCTC**	-289 to -21	19	60
Neuregulin-1	NRG1/ 8	Fw: TTAAAGGAGTATGGAGTAGAGGAGG,<br>**Rev_i: TCCTTTCCCCATACTACTAC**,<br>Rev_o:AATAACAATCCTTTATATAAAATAATACTA	-404 to -37	28	70
Neurexin	NRXN1/ 2	Fw_o: AAGAGAGGGTGGTGATAA,<br>**Fw_i: GGTGGTGATAAGTTAGAAG**,<br>Rev: GAATACTATCCTTCTAAAAAAAA	+121 to +239	10	70
Synaptophysin	SYP/ X	Fw: GTGAGATTGTGGTAT,<br>**Rev_i: CAACAACAACATCAACAATACAAAAA**,<br>Rev_o: TACCAAACCCTAACCACCAC	-191 to +6	20	70
Polysialic Acid Transferase 2 (T1)	ST8SIA2/15	Fw_o: TGGGGGGTATTATTTTTT<br>Fw_i: TTATTGATTTTAGGGTTAT<br>**Rev: ATCTTTATCTCCATTTAT**	-380 to +9	39	48
Polysialic Acid Transferase 4	ST8SIA4/5	Fw: TAGTATTGTGAGTTTTTTTTTT<br>**Rev: CCACCTCTTCCTAATAA**	-149 to +336	28	56
NCAM (Neural Cell Adhesion Molecule)	NCAM1/ 11	Fw_a: AAGGAAGGTTGGGTAGTAGGA,<br>Fw_i: GGGTAGAAGTGTGAAAAG,<br>**Rev_i: ACAAACCTAAAAACAAACAAT**,<br>Rev_a: TCCCAAAAAAAACAAAATCCAA	-95 to +185	22	46

Supplemental Table 1: List of bisulfite-primers. Primers used for the sequencing PCR are highlighted in bold letters.

Gene	Gene symbol	Primer Sequences	Type	Mapped Transcripts (T)	Location E= Exon
Tau (Microtubule-associated protein Tau)	MAPT	Fw: 5’GACTATCAGGTGAACTTT3’<br>Rev: 5’GCCAGCGTCCGTGT3’	TG	All T	E1-E2,<br>E2
Neurofilament-Heavy polypeptide	NEFH	Fw: 5`GAGTGGTTCCGAGTGAG3`<br>Rev: 5`CTGAATGGCTTCCTGGTAG3`	TG	The only T	E1-E2<br>E2-E3
Canabinoid Receptor 1	CNR1	Fw: 5’GATGCGAAGGGATTGCC3’<br>Rev: 5’CAGGAGGTCAGTGGTG3‘	TG	T NM_016083.4 (predominant)	E1-E4,<br>E4
Neuregulin-1	NRG1	Fw: 5´CTAAACTTTCTACATCTACA3’<br>Rev: 5’TAAACTCATTTGGGCACTT3’	TG	T NM_013959.3 (especially important in neurons)	E8-E10e<br>E10e-E12b
Neurexin	NRXN1	Fw: 5`CCATTAGCCAGACCACAGAT3`<br>Rev: 5`CACAGGGCGGCAGCG3`	TG	All T	E23-E24<br>E25
Synaptophysin	SYP	Fw: 5`GCAGCGGTGGCAGTGGC3`<br>Rev: 5`GGACGGGGTAAGAGAGGGG3`	TG	The only T	E3,<br>E7
Dysbindin1 (Dystrobrevin Binding Protein1)	DTNBP	Fw: 5`GTAGTGGGAGTGCGGG3`<br>Rev: 5`GTCCTGGGTTTGCTTTTCA3`	TG	T NM_032122.4 and T NM_183040.2 (coding)	E1<br>E2-E3
Reelin	RELN	Fw: 5`ATTAGAGCCCCTTCCA3`<br>Rev: 5`CCAGTTGCCTGTGTC3`	TG	All T	E9-E10<br>E10
Glyceraldehyde-3-Phosphate Dehydrogenase	GAPDH	Fw: 5`CGGATTTGGTCGTATTGGGCG3`<br>Rev: 5`GCTCCTGGAAGATGGTGATGGG3`	RG	All T	E2-E3<br>E4-E5
Succinate Dehydrogenase Complex, subunit A	SDHA	Fw: 5`ATGG AAGG TCTC TGCG ATA3`<br>Rev: 5`CCGT AGCC TCCT GTGG3`	RG	All T	E5-E6<br>E6-E7
Ubiquitin C	UBC	Fw: 5`AAG GAC ATT TTA GGA CGG GA3`<br>Rev: 5`TTACCAGTCAGAGTCTTCACGA3`	RG	The only T	E1<br>E2

Supplemental Table 2: Primer sequences for real-time measurement of different candidate genes. Whenever possible, primers were designed to span an exon-exon border to exclude amplification of genomic DNA residues. This table also depicts every candidate gene´s involved exons and amplified transcripts (according to NCBI Reference Sequence for mRNA, NM). TG indicates target genes, and RG indicates the utilized reference genes. The most stable reference gene as revealed by geNorm analysis for our experiment setting and type of cells, was GAPDH.

Gene	Optimal PCR Temperature (°C), (Y°C)	Primer efficiency	Length of amplicon (Bp)	Chromosome
Tau (Microtubule-associated protein Tau)	55.5	1.958	152	17
Neurofilament-Heavy polypeptide	55.5	1.994	225	22
Canabinoid Receptor 1	58.1	1.943	136	6
Neuregulin-1	50.0	1.990	156	8
Neurexin	61.6	1.973	214	2
Synaptophysin	61.6	1.900	330	X
Dysbindin1 (Dystrobrevin Binding Protein1)	55.5	1.973	228	6
Reelin	58.1	1.978	219	7
Glyceraldehyde-3-Phosphate Dehydrogenase	64.4	1.966	210	12
Succinate Dehydrogenase Complex, subunit A	63.5	1.983	166	5
Ubiquitin C	60.0	1.980	266	12

Supplemental Table 3: Additional information concerning real-time PCR.

Secondary Antibody	Company	Order no.	Dilution
Cy 3, goat anti-mouse	Life Technologies	A10521	1:500
Cy 3, goat anti-rabbit	Millipore	AP132C	1:400
Alexa 488, goat anti-rabbit	Life Technologies	A11008	1:250

Supplemental Table 4: Secondary antibodies used for ICC.

Primary Antibody	Company	Order No.	Dilution	Sec. Antibody
Tau, rabbit anti-human	Millipore	MAB10417	1:100	Cy3 (goat anti rabbit)
ß-Tubulin, mouse anti-human	Millipore	05-559	1:500	Cy 3 (goat anti mouse)
MAP 2, rabbit anti-human	Millipore	AB5622	1:500	Alexa 488
DRD2, rabbit anti-human	Novus Biological	NB600-1261	1:1000	Alexa 488
CB 1, rabbit anti-human	Calbiochem	209550	1:100	Alexa 488
mGluR1, rabbit anti-human	Thermo Scientific	PA1-4516	1:100	Alexa 488
NMDAR1, rabbit anti-human	Pierce	PA3-102	1:300	Alexa 488
Grik, rabbit anti-human	Thermo Scientific	PA5-20447	1:100	Alexa 488
PSD 95, rabbit anti-human	Millipore	04-1065	1:70	Alexa 488
Synaptophysin, rabbit anti-human	Abcam	Ab23754	1:500	Alexa 488

Supplemental Table 5: Primary antibodies used for ICC.

Target	Species	Company	Order-No.	Dilution
ß-Tubulin	Ms	Millipore	05-559	1:1000
DRD2	Rb	Abcam	Ab85367	1:1000
Grik	Rb	Abcam	Ab67317	1:300
NMDAR1	Ms	Abcam	Ab134308	1:300
Syp	Rb	Invitrogen	MA5-14532	1:300
PSD 95	Rb	Abcam	Ab18258	1:300

Supplemental Table 6: Western Blot antibodies

Suppl. Table 7A: Methylation Rates					
**Mean, SD**					
**Gene/Substance**	**RA 50**	**Co**	**THC 20**	**An 70**	**HU20**
**MAPT**	26.8 (1.6)	28.1 (1.4)	27.9 (1.8)	26.4 (2.2)	28.1 (1.9)
		F(1, 1023)=0.34	F(3,1513)=0.05	F(3,1144)=0,22	F(3,1222)=0.24
**ß-Aktin**	80.8 (3.2)	76.2 (2.8)	77.3 (2.7)	75.6 (3.2)	75.9 (2.9)
		F (1, 170)= 1.42	F (3, 318)= 1.04	F (3, 315)= 1.17	F (3, 310)= 0.6
**MAP2**	37.0 (7)	38.9 (5)	38 (5)	41.9 (9)	40.5 (6.2)
		F(1, 61)= 0.05	F (3, 143)= 0.018	F (3, 94)= 0.16	F(3,122)= 0.05
**DRD2**	6.5 (0.5)	4.7 (0.5), p<0.05	4.3 (0.6), p<0.05	4.3 (1), p<0.05	4.5 (0.7), p<0.05
		F(1,1295)=5.23, p≤0.001	F(3,1295)=5.23, p≤0.001	F(3,1431)=6.43, p≤0.001	F(3,1691)=5.39, p≤0.001
**CB1**	9.4 (2.2)	2.2 (3.7), p<0.05	7.6 (2.1)	8.4 (2.6)	9.1 (2.6)
		F (1, 66)= 4.58, p<0.05	F (3, 126)= 0.19	F(3, 125)= 1.09	F (3,104)=2.2
**NRG**	7.6 (3.2)	4.6 (4.2), p<0.05	6.4 (2.4)	4.6 (5.2)	7.6 (3.1)
		F(1, 50)= 11.47, p≤0.001	F(3,143)= 0.32	F(3, 104)= 0.47	F(3,143)= 1.15
**Nx**	2.5 (0.6)	0 (0.8)	1 (0.6)	0.4 (0.8)	1.6 (0.8)
		F(1,54)=0.55	F(3,112)=1.99	F(3, 98)=0.77	F(3, 92)=0.548
**Syph**	12.1 (2.2)	4.7 (2.0), p<0.05	3.3 (1.4), p<0.001	2.3 (1.6), p<0.001	3.3 (1.5), p<0.05
		F(1, 80)=6.33, p<0.05	F(3, 165)= 5.73, p≤0.001	F(3, 137)= 7.68, p≤0.001	F(3,147)=5.77, p≤0.001
**ST8SIA2**	2 (0.2)	2.7 (0.2), p<0.05	1.9 (0.2)	2 (0.2)	2.6 (0.3)
		F(1,1269)=6.68, p≤0.001	F(3,1929)=1.25	F(3,1271)=3.95	F(3,1732)=2.19
**ST8SIA4**	6.4 (0.5)	6.5 (0.6)	4.6 (0.5), p<0.05	4.1 (0.7), p<0.05	5.6 (0.5)
		F(1,458)=0.029	F(3,780)=3.72; p=0,01	F(3,532)=4.28, p<0.05	F(3,682)=0.86
**NCAM**	5.1 (0.5)	5.0 (0.6)	5.3 (0.6)	4.5 (0.7)	5.2 (0.8)
		F(1, 500)= 0.02	F(3,783)=0.13	F(3,557)=0.43	F(3,583)=1.2

Supplemental Table 7A: Due to the high number of CpGs investigated in every gene, the mixed linear model (based on ANOVA) was used to analyze group differences of mean methylation rates. Respective substance classes were always analyzed with the RA50-treated cell control group. For each analysis, mean values (S.E.M.) of the pairwise comparisons to RA50 are given in the first row of every gene. Respective significance levels are shown in the case of significant group differences (also indicated by a grey background). In the case of non-significant differences, there is no p-value and the background is white to keep it as simple as possible for a better overview. In the second row (below), also the respective group effects characterized by F-values and degrees of freedom as well as the significance level are shown for better assessment of the data.

**Suppl. Table 7B: Real Time Data**					
**Mean, 95 % CI low/high**					
**Gene/Substance**	**RA**	**Co**	**THC 20**	**An 70**	**Hu 20**
**MAPT**	1 (0.36/2.8)	1.63 (0.41/2.43)	0.6 (0.35/1.04)	0.61 (0.51/0.72)	1.27 (0.68/2.39)
**NEFH**	1 (0.45/2.24)	3.21 (0.33/3.5)	0.53 (0.17/1.63)	1.3 (0.79/2.39)	0.95 (0.41/2.19)
**CNR1**	1 (0.3/3.3)	0.19 (0.1/1.57), p<0.05	0.56 (0.32/0.97)	0.96 (0.26/3.52)	1.09 (0.55/2.15)
**NRG1**	1 (0.37/2.68)	0.46 (0.3/1.84)	0.34 (0.18/0.64)	0.29 (0.15/0.53)	0.27 (0.13/0.54)
**Nx**	1 (0.39/2.58)	0.31 (0.2/1.9), p<0.05	0.52 (0.33/0.81)	0.52 (0.29/0.91)	0.46 (0.29/0.74)
**SYP**	1 (0.48/2.09)	1.31 (0.38/2.64)	1.15 (0.58/2.27)	1.77 (0.87/3.6)	1.66 (1/2.75)
**Dysb**	1 (0.49/2.06)	0.3 (0.24/4.17), p<0.05	1.02 (0.46/2.26)	0.68 (0.38/1.21)	0.68 (0.53/0.88)
**RELN**	1 (0.28/3.56)	0.24 (0.2/3.72)	0.17 (0.03/0.92)	0.44 (0.12/1.58)	0.11 (0.04/0.29)

Suppl. Table 7B: RNA expression level in relation to 50 µM RA: Means (95% Confidence Interval low/high). Grey background: significantly different in comparison to RA 50, white background: non-significant differences in comparison to RA50. qBase; Mann-Whitney-U-Test.

Suppl. Table 7C: Western Blot Data			
**Mean, SD**			
**Target/Substance**	**RA50**	**Co**	**THC**
**ß-Tubulin**	1 (0)	0.58 (0.3)	0.53 (0.37)
**DRD2**	1 (0)	0.42 (0.02)	0.5 (0.01)
**Grik**	1 (0)	0.52 (2.9)	0.66 (0.09)
**NMDAR1**	1 (0)	0.37 (0.01)	0.61 (0.02)
**SYP**	1 (0)	0.68 (0.38)	0.79 (0.14)
**PSD 95**	1 (0)	1.8 (0.48)	1.34 (0.07)

Suppl. Table 7C: Protein expression level in relation to 50µM RA treated cells. Mean (SD). Differences were non-significant. Graph Pad Prism; One-way ANOVA.
